# Marine-derived fungi from the genus *Aspergillus* (Ascomycota) and their anticancer properties

**DOI:** 10.1080/21501203.2024.2402309

**Published:** 2024-11-04

**Authors:** Jessica Mélanie Wong Chin, Rajesh Jeewon, Abdulwahed Fahad Alrefaei, Daneshwar Puchooa, Theeshan Bahorun, Vidushi S. Neergheen

**Affiliations:** a Biopharmaceutical Unit, Center for Biomedical and Biomaterials Research (CBBR), University of Mauritius, Reduit, Mauritius; b Department of Agricultural and Food Science, Faculty of Agriculture, University of Mauritius, Reduit, Mauritius; c Department of Health Sciences, Faculty of Medicine and Health Sciences, University of Mauritius, Reduit, Mauritius; d Department of Zoology, College of Science, King Saud University, Riyadh, Saudi Arabia; e Department of Biosciences and Ocean Studies, Faculty of Science, University of Mauritius, Reduit, Mauritius

**Keywords:** *Aspergillus*, cytotoxic, marine fungi, metabolites

## Abstract

Marine fungi are promising sources of bioactive natural products. The harsh marine conditions favour the production of natural products with unique structures and functions. The different classes of bioactive metabolites produced by these marine fungi can exhibit cytotoxic, apoptotic, anti-proliferative, antiangiogenic, and autophagy inducing effects on a plethora of cancer cell lines. This review, based on research articles that have been published from 2002 to 2023, provides a concise overview of the anticancer properties of metabolites from marine *Aspergillus* fungal species. A total of 204 papers are reviewed and 208 most active cytotoxic molecules are reported from *Aspergillus*. The source as well as the growth medium utilised for the production of cytotoxic metabolites are listed. The mechanism of action of some compounds, which could be used as potential drugs, is also reported. These fungi, under optimal growth conditions, have immense potential as anticancer agents, produce novel metabolites with specific structures that can kill a panel of human cancer cells. However, there is a dire need for more clinical trials and understanding of the mechanisms of action of pharmacologically active constituent. Research should also target how to improve culture methods and perform clinical research on human subjects with more scientific reproducibility.

## 1. Introduction

Obligate marine fungi are confined to growth and sporulation only in marine or estuarine habitats whereas facultative marine fungi originate from freshwater or terrestrial habitats and are fitted for growing and producing spores in marine environments (Kohlmeyer and Kohlmeyer 1979). A reviewed and broader definition of marine fungus that takes into account several parameters, according to Pang et al. (2016) is “any fungus that is recovered repeatedly from marine habitats because: 1) it is able to grow and/or sporulate (on substrate) in marine environments; 2) it forms symbiotic relationships with other marine organisms; or 3) it is shown to adapt and evolve at the genetic level or be metabolically active in marine environments”.

Marine fungi have been found growing in different environments and are associated with various hosts. Several factors affect fungal diversity including availability and accessibility of substrates, water temperature, salinity, pH and depth, mycelium growth rate, and competition with other organisms (Bunbury-Blanchette and Walker 2019). Marine fungi that exist in the saline conditions of the sea act as saprobes, pathogens, or symbionts. Many authors have isolated marine fungi from a plethora of materials from different depths of the sea with high or low oxygen levels (Rédou et al. 2015; Ogaki et al. 2020), hydrothermal vents (Tao et al. 2018), wood (Li et al. 2018a; Björdal and Dayton 2020; Shen et al. 2022), sediments (Khusnullina et al. 2018), sand (Gomes et al. 2008), algae and seagrasses (Venkatachalam 2015; Ettinger et al. 2020; Pasqualetti et al. 2020), corals (Góes-Neto et al. 2019), mangroves (Lee et al. 2020), sponges and living marine invertebrates (Swe et al. 2008; Frank et al. 2019; Wong Chin et al. 2021), calcareous shells of molluscs, crab exoskeleton, cuttlefish endoskeletons and feathers (Ananda et al. 1998; Gleason et al. 2017), and from the gut of marine isopods (Li et al. 2016d, 2023) as well as from other decayed susbtrates (Devadatha et al. 2018a, 2018b).

Cancer can be defined as the abnormal growth and division of cells without control (National Cancer Institute 2020; Sonnenschein and Soto 2020; Kato et al. 2021). Cancer arises when changes in the proto-oncogenes, tumour-suppressor genes, and DNA repair genes that control cell growth and division happen due to genetic inheritance or environmental conditions like chemicals in tobacco, radiation, and UV rays (Lagoa et al. 2020). In 2020, cancer was among the leading causes of death, with 10.0 million deaths worldwide (WHO 2022). Lung cancer was the most fatal (1.8 million deaths), followed by colon and rectum (916,000 deaths), liver (830,000 deaths), stomach (769,000 deaths), and breast (685,000 deaths) (WHO 2022). Several treatments are used including immunotherapy, radiotherapy, and chemotherapy, but cancer is still a leading disease with high mortality rates and is a global public health issue.

Marine microorganisms produce anticancer natural products and represent an untapped source for discovering novel natural drug-like molecules. There are many examples of cytotoxic natural products from marine fungi, including novel structures with potential bioactivity (Ameen et al. 2020; Law et al. 2020; Petersen et al. 2020; Aullybux et al. 2021; Karthikeyan et al. 2022). Reported metabolites can be classified into the five major classes terpenoids and sterols, peptides, phenolics, alkaloids, and polyketides (Hasan et al. 2015; Jeewon et al. 2019). The discovery of taxol from the fungal endophyte *Taxomyces andeanae* of the Pacific yew *T. brevifolia* by Stierle et al. (1993) has shown that fungi are producers of cytotoxic metabolites. Those associated with sponges produce terpenes, alkaloids, peptides, lactones, and steroids with therapeutic effects. Six new sorbicillinoid derivatives were isolated from a sponge-derived fungus *Trichoderma reesei*. 15-hydroxy-bisvertinol was the identified compound that showed cytotoxicity against A549, MCF-7, and HCT116 with IC_50_ values of 5.1, 9.5, and 13.7 µmol/L, respectively (Rehman et al. 2020). Endophytes derived from macro-algae are also capable of producing cytotoxic compounds as reported by Jiang et al. (2020). The solid culture of *Penicillium chrysogenum* LD-201810 produced a new hydroxyphenylacteic acid derivative, (2’*R*)-westerdijkin A with IC_50_ value of 22.0 µmol/L against HepG2 cell line. It was found that the compound was able to induce apoptosis due to the fragmented/condensed nucleus and apoptotic body formation. The marine fungi *Paradendryphiella salina* produced (-)-hyalodendrin on seawater MEA with IC_50_ values of 0.4 µg/mL, 0.2 µg/mL, and 0.5 µg/mL against MCF7, MCF7-Sh-WISP2, and 3T3-F442A cells. This compound was able to induce changes in the phosphorylation of p53 and alter the expression of the proteins HSP60, HSP70, and PRAS40 (Dezaire et al. 2020). The active fraction of the novel marine sediment-derived *Penicillium* sp. ArCSPf, displayed significant cytotoxic properties against MCF-7 breast cancer cells (IC_50_ = 22.79 µg/mL) (Farha and Hatha 2019). Even deep-sea fungi are capable of producing cytotoxic compounds. Li et al. (2022b) isolated the marine *Penicillium* sp. LXY140-R and *Penicillium* sp. LXY140-3, which produced anti-proliferative compounds against HCT-116, A549, and Bel-7402 cell lines. Their low IC_50_ against the cancer cell lines justifies interest to probing further into their potential mechanism of action as well as preclinical and clinical studies since the American National Cancer Institute (NCI) considers an IC_50_ < 20 µg/mL or 10 µmol/L after 48 h or 72 h, as interesting candidate for drug prospecting (Canga et al. 2022).

The genus *Aspergillus* (Trichocomaceae, Eurotiales, Ascomycota) consists of asexual spore-forming fungi (Lee et al. 2016). It is widely distributed in the terrestrial and marine habitats and produces various mycotoxins (Orfali et al. 2021; Youssef et al. 2021). Marine *Aspergillus* have received a lot of attention as they produce bioactive compounds with antimicrobial, cytotoxic, anti-oxidant, anti-inflammatory, antiviral, insecticidal, and neuroprotective properties. Different secondary metabolites, ranging from alkaloids, fatty acids, steroids, terpenoids, polyketones, peptides, butenolides, and lactones, have been recovered from these isolates (Orfali et al. 2021). These metabolites represent lead compounds that have to be exploited in the pharmaceutical industry. An interesting anticancer drug in phase III clinical trial is Plinabulin. It was derived from the natural product halimide, which was produced by a marine *Aspergillus* sp. (Saeed Abdullah et al. 2021). Hence, this review paper will highlight the chemical diversity of cytotoxic compounds produced by marine *Aspergillus* species and the mechanisms of action of some of these compounds. The compounds were classified based on the sources of the fungi. It also emphasises their potential as anticancer lead compounds.

## 2. Methodology

**Figure 1.**
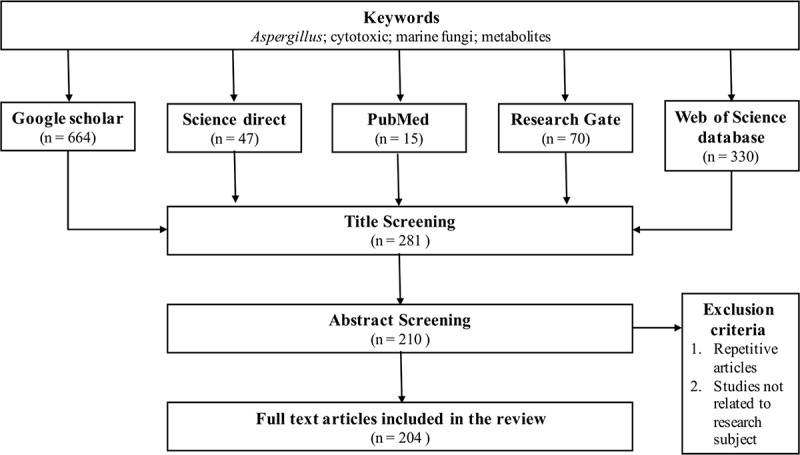
Schematic representation of the method used to retrieve data.

Literature search was performed on Google Scholar, ScienceDirect, PubMed, ResearchGate, and Web of Science databases. The search terms were as follows: anticancer, marine fungi, cytotoxicity, and *Aspergillus*. Articles from 2002 to 2023 were considered and were selected according to Figure 1. The most active compounds (IC_50_ < 20 µg/mL or 10 µmol/L after 48 h or 72 h), which have strong cytotoxic potentials, were drawn using ChemDraw.

## 3. Metabolites isolated from *Aspergillus* genus

### 3.1. *Compounds from* Aspergillus *associated with marine sponges*

Three metabolites, bicoumanigrin A, aspernigrins A, and B, were isolated from the fungus *Aspergillus niger* associated with Mediterranean sponge *Axinella damicornis*. They had moderate cytotoxicity against a panel of different human leukaemia and carcinoma cell lines at 50 µg/mL (Hiort et al. 2004). Liu et al. (2009) isolated drimane sesquiterpenoids, mono(6-strobilactone-B) ester of (*E,E*)-2,4-hexadienedioic acid (**1**), (6-strobilactone-B) ester of (*E,E*)-6-oxo-2,4-hexa-dienoic acid (**2**), RES-1149-2 (**3**), from the fungus *Aspergillus ustus*. This showed cytotoxicity against L5178Y, HeLa, and PC12 cells, with IC_50_ ranging from 0.6 to 7.2 µg/mL (Table 1). Six metabolites were recovered from *Aspergillus versicolor*, namely sterigmatocystin (**4**), averantin (**5**), methyl-averantin (**6**), averufin (**7**), nidurufin (**8**), and versiconol (**9**). These had cytotoxic activities against human cancer cell lines A549, SK-OV-3, SK-MEL-2, XF-498, and HCT-15 with low IC_50_ values of 0.41–4.61 µg/mL (Lee et al. 2010). From the same fungus, fellutamide C (**10**) and F (**11**) were also isolated by Lee et al. (2011b). Fellutamide C (**10**) has strong cytotoxic properties against the five cancer cell lines used with IC_50_ values ranging from 0.13 to 1.81 µg/mL.

**Table 1.** Compounds isolated from *Aspergillus* species associated with marine sponges.

Sponge species	Region/country	Media	Fungi		Compound	Cancer cell line	Activity (IC_50_)	Reference
*Suberites domuncula*	Adriatic Sea	Biomalt agar, barley-spelt solid medium	*Aspergillus ustus*		Mono(6-strobilactone-B) ester of (*E,E*)-2,4-hexadienedioic acid (**1**), (6-strobilactone-B) ester of (*E,E*)-6-oxo-2,4-hexa-dienoic acid (**2**), RES-1149-2 (**3**)	L5178Y, HeLa, PC12	0.6 to >10 µg/mL Positive control (Kahalalide F): 6.3 µg/mL	Liu et al. (2009)
*Petrosia* sp.	Jeju Island, Korea	Malt media	*Aspergillus versicolor*		2,4-dihydroxy-6-((*R*)-4-hydroxy-2-oxopentyl)-3- methylbenzaldehyde, sterigmatocystin (**4**), dihydrosterigmatocystin, averantin (**5**), methyl-averantin (**6**), nidurufin (**8**), averufin (**7**), versiconol (**9**)	A549, SK-OV-3, SK-MEL-2, XF-498, HCT-15	0.41 to >30.0 µg/mL Doxorubicin: 0.002–0.034 µmol/L	Lee et al. (2010)
*Petrosia* sp.	Jeju Island, Korea	Malt media	*Aspergillus versicolor*		Fellutamide C (**10**), F (**11**)	A549, SK-OV-3, SK-MEL-2, XF498, HCT15	0.13–18.42 µg/mL Doxorubicin: 0.01–0.18 µmol/L	Lee et al. (2011b)
*Xestospongia testudinaria*	South China sea	Glucose yeast extract peptone	*Aspergillus* sp.		Disydonols A (**12**), C (**13**)	HepG2, Caski	2.91–12.40 µg/mL	Sun et al. (2012)
*Cinachyrella australiensis*	South China Sea	–	*Aspergillus insulicola* MD10-2		Insulicolide A (**14**)	H-406	6.9 µmol/L	Zhao et al. (2016)
*Agelas oroides*	Aliaga-Izmir coast of Aegean Sea, Turkey	Rice medium, modified Czapek medium	*Aspergillus carneus*		Isopropylchaetominine (**15**), sterigmatocystin (**16**), asteltoxin E (**17**), versicolorin C, nidurufin, norsolorinic acid, O-demethylsterigmatocystin C	L5178Y	0.2–25 µmol/L	Özkaya et al. (2018)
Sponge	–	–	*Aspergillus violaceus* WZXY-m64-17		Violaceimide A (**18**), B (**19**), E	U937, HCT-8, MCF-7, Vero	1.5 ± 0.28 to >100 µmol/L Paclitaxel: 0.3 ± 0.01 µmol/L	Yin et al. (2018)
Sponge	Xuwen County, Guangdong Province, China	Rice medium	*Aspergillus* sp. SCSIO XWS03F03		Misszrtine A (**20**)	HL60, A549, HT29, SK-BR-3, MCF-7, LNCaP	3.1 to >30 µmol/L	Zhou et al. (2018)
*Agelas oroides*	Sıgaçık-Izmir, Turkey	Rice medium	*Aspergillus ochraceus*		Viomellein (**21**), ochratoxin B (**22**)	A2780, l5178Y	5.0 µmol/L, 3.0 µmol/L, 5.3 µmol/L	Frank et al. (2019)
Sponge	–	–	*Aspergillus* sp. SCSIO 41018		Asterriquinones I, J (**23**), K, B1 (**24**)	K562, BEL-7042, SGC-7901, A549, HeLa	8.5 ± 0.17 to >30 µmol/L Paclitaxel: 0.7 ± 0.03 to 3.3 ± 0.22 µmol/L	Guo et al. (2019)
*Haliclona* sp.	Lingshui, Hainan Province, China	Potato dextrose broth	*Aspergillus* sp. LS34		Asperspin A, asperther A, gibellulin B, daldinin C, oxalicumone A (**25**)	CCRF-CEM, K562, HCT-116, MDA-MB-453, COR-L23	1.22 ± 0.05 to 29.28 ± 0.75 µmol/L	Li et al. (2019b)
Sponge	Pramuka Island, Indonesia	Rice medium	*Aspergillus* sp.		Physcion, 2-(20,3-epoxy-10,30,50-heptatrienyl)-6-hydroxy-5-(3-methyl-2-butenyl) benzaldehyde (**26**)	PANC	6.0 µmol/L, 1.7 µmol/L	Abdel-Naime et al. (2020)
Sponge	Xisha islands, South China Sea	Mannitol, glucose, maltose, yeast extract, glutamate, corn syrup	*Aspergillus candidus* OUCMDZ-1051		4-*O*-methylcandidusin A (**27**)	N87, A673, MV4-11, K562, A549, BT474, H1299, HUCCT1, MDA-MB-468, H1975, HL-60, Karpas299, U87, A431, U251, HCC1954, MCF-7, MKN-45, DU145, SPC-A1, HCT116, MDA-MB-231, 143B, B16F10, H2228, Hep3B	1.84 ± 0.02 to >100 µmol/L Doxorubicin: 0.02 ± 0.01 to >100 µmol/L	Wang et al. (2020a)
*Stylissa* sp.	Nha trang Bay, Vietnam	Bennett’s medium		*Aspergillus flocculosus* 01NT.1.1.5	(5*R*,6*S*,16*R*,3*E*)-5,6-dihydroxy-16-methyloxacyclohexadec-3-en-2-one (**28**), bekeleylactone E (**29**)	PC-3, HCT-15, MDA-MB-231, ACHN, NCI-H23, NUGC-3	1.1–3.6 µmol/L Adriamycin: 0.12–0.17 µmol/L	Anh et al. (2021)
*Neopetrosia chaliniformis*	Mandeh Island, West Sumatra, Indonesia	Rice medium	*Aspergillus nomius* NC06		Oxisterigmatocystins J (**30**), K, L, aspergillicin A (**31**)	HT29	1.63–988.05 µmol/L Taxol: 0.48 µmol/L	Artasasta et al. (2021)
*Agelas oroides*	Aliaga-Izmir coast of Aegean Sea, Turkey	Rice medium	*Aspergillus carneus*		Averufanin (**32**), nidurifin, versicolorin, averufin, arugosin C	MCF7, MDA-MB-213, OVCAR3, OVSAHO, KURAMOCHI, HGRC1	0.28 ± 0.1 to >40 µmol/L	Demirel et al. (2023)
Sponge	Nha Trang bay	Rice yeast extract medium	*Aspergillus* sp. 1901NT-1.2.2		Vismione E (**33**)	PC-3, MCF-7, MCF-10A, H9c2	9.0 ± 0.4 to 69.8 ± 8.0 µmol/L	Girich et al. (2023)

The five compounds, aspergiterpenoid A, (-)-sydonol, (-)-sydonic acid, (-)-5-(hydroxymethyl)-2-(2′,6′,6′-trimethyltetrahydro-2 H-pyran-2-yl)phenol, and (*Z*)-5-(hydroxymethyl)-2-(6′-methylhept-2′-en-2′-yl)phenol, isolated from *Aspergillus* sp. had weak cytotoxicity (IC_50_ > 50 µg/mL) against HL-60 and A-549 cells (Li et al. 2012). Two new phenolic bisabolane sesquiterpenoid dimers, disydonols A (**12**) and C (**13**), showed cytotoxicity against HepG2 and Caski human cancer cell lines, with IC_50_ ranging from 2.91 to 12.40 µg/mL. These compounds were isolated by Sun et al. (2012) from the fungus *Aspergillus* sp. associated with the sponge *Xestospongia testudinaria*. Various *Aspergillus terreus* strains and one *Aspergillus ochraceus*, isolated from sponges, had strong cytotoxic activities (IC_50_ < 50 µg/mL) against A549, A-375, Bel-7402, and MRC-5 (Yu et al. 2012). The mycelium extract from the marine fungus *Aspergillus unguis* RSPG_204 showed strong cytotoxicity against MCF7 with IC_50_ of 9.98 µg/mL. The broth and mycelium extract showed good cytotoxicity against HeLa, HepG2, and HCT-116 (Abd El-Hady et al. 2014). Terrein was isolated from the sponge-associated *Aspergillus terreus* strain PF-26 (Chen et al. 2014b). This compound inhibited the growth of the human epithelial ovarian cancer cells at a concentration of 15 mg/L.

A new hexacyclic peptide, similanamide, produced by *Aspergillus similanensis* KUFA 0013, showed cytotoxicity against three cancer cell lines, MCF-7, NCI-H460, and A373. The IC_50_ were 125 ± 0 µmol/L, 17.50 ± 3.55 µmol/L, and 115 ± 7.07 µmol/L, respectively (Prompanya et al. 2015). The fungus *Aspergillus similanensis* KUFA 0013 was isolated from the sponge *Rhabdermia* sp. Its crude extract showed cytotoxic properties against a range of cancer cells, namely, HepG2, HT29, HCT116, U251, A549, A375, and MCF7 with IC_50_ > 200 µg/mL (Ramos et al. 2015). The fungus *Aspergillus insulicola* MD10-2, isolated from the South China sea sponge *Cinachyrella australiensis*, produced nitrobenzoyloxy-substituted sesquiterpenes with cytotoxic properties against the human cancer cell line H-460. The lowest IC_50_ value obtained with insulicolide A (**14**) was 6.9 µmol/L (Zhao et al. 2016). The crude extract of *Aspergillus nomius* was cytotoxic to WiDr cells but was not cytotoxic to normal Vero cells, at a concentration of 100 ppm (Ade Artasasta et al. 2017). Two new metabolites were isolated by Liu et al. (2017b) from the fungus *Aspergillus sydowii* J05B-7F-4. The compounds diorcinolic acid and β-D-glucopyranosyl aspergillusene A had mild cytotoxicity against the human nasopharyngeal carcinoma cells (KB), human liver cancer cells (HepG2) and human colon cancer cells (HCT-116). The IC_50_ ranged from 50 to 70 µmol/L.

Buttachon et al. (2018) isolated the six cytotoxic compounds, petromurin C, kumbicin B, candidusin D, 2’-oxoasterriquinol D methyl ether, preussin, and preussin C, from the fungus *Aspergillus candidus* KUFA0062, obtained from the sponge *Epipolasis* sp. These compounds had cytotoxic activities against eight cancer cell lines, HepG2, HT29, HCT116, A549, A375, MCF-7, U-251, and T98G. The IC_50_ of the compounds ranged from 12.3 to 212.5 µmol/L. The extract of *Aspergillus versicolor* MERVA29 had cytotoxic properties against HepG2 and Caco-2 cells, with IC_50_ > 200 µg/mL (El-Gendy et al. 2018). The extract of *Aspergillus* sp., isolated from the marine sponge *Haliclona fascigera*, had cytotoxic activities against the cancer cell lines Hela, WiDr, T47D, and Vero. The IC_50_ obtained from the crude extract ranged from 38.21 to 598.89 ppm (Handayani et al. 2018a). Isopropylchaetominine (**15**), sterigmatocystin (**16**), and asteltoxin E (**17**), isolated from the fungus *Aspergillus carneus*, associated with sponge *Agelas oxoides*, showed strong cytotoxicity against the mouse lymphoma cell-line L5178Y with IC_50_ values of 0.4, 0.3, and 0.2 µmol/L, respectively. In addition, four other compounds, versicolorin C, nidurufin, norsolorinic acid, and O-demethylsterigmatocystin C, isolated from the same fungus had moderate cytotoxicity against L5178Y with IC_50_ values of 20, 9, 25, and 10 µmol/L, respectively (Özkaya et al. 2018). The cytotoxic compounds violaceimide A (**18**) and B (**19**) were isolated from the fungi *Aspergillus violaceus* WZXY-m64-17. These caused the cell death of U937, HCT-8, MCF-7, and Vero cancer cells, with IC_50_ ranging from 1.5 ± 0.28 to >100 µmol/L (Yin et al. 2018). Zhou et al. (2018) isolated the fungus *Aspergillus* sp. SCSIO XWS03F03 from a sponge in China. Its compound, misszrtine A (**20**), had cytotoxic activity against HL60 and LNCaP cells at 3.1 µmol/L and 4.9 µmol/L, respectively.

The extract of the fungus *Aspergillus ochraceus* was more cytotoxic against WiDr cells but not Vero cells (Aminah et al. 2019). The fungus *Aspergillus flavus* Af/MMA produced the compound aurasperone E which had cytotoxic properties against Ehrlich ascites carcinoma cells (El Awady et al. 2019). *Aspergillus ochraceus* produced the two secondary metabolites viomellein (**21**) and ochratoxin B (**22**). These had strong cytotoxicity against human ovarian carcinoma A2780 and L5178Y with IC_50_ of 5.0 µmol/L and 3.0 µmol/L against A2780 and 5.3 µmol/L against L5178Y (Frank et al. 2019). Asterriquinones I, J (**23**), K, and B1 (**24**) purified from the fungus *Aspergillus* sp. SCSIO 41018, had cytotoxic activities against K562, BEL-7042, SGC-7901, A549, and Hela cell lines. The IC_50_ ranged from 8.5 ± 0.17 µmol/L to >30 µmol/L (Guo et al. 2019). A fungal strain, *Aspergillus* sp. LS34, was isolated from the sponge *Haliclona* sp. in Lingshui, Hainan Province, China. The compound oxalicumone A (**25**) showed pronounced cytotoxicity against CCRF-CEM and K562 with IC_50_ of 1.22 ± 0.045 µmol/L and 9.58 ± 0.19 µmol/L, respectively. Four other compounds produced by this fungus also showed weak cytotoxicity. These were asperspin A, asperther A, gibellulin B, and daldinin C which killed the cancer cells HCT-116, CCRF-CEM, K562, MDA-MB-453, and COR-L23 (Li et al. 2019b). Butyrolactone I, obtained from the culture of *Aspergillus terreus* SCSIO 41008, had cytotoxic properties against U87 and HT22 cells (Luo et al. 2019b). The fungus *Aspergillus versicolor* SCSIO 41016, produced protuboxepin C, G that had cytotoxic properties against the cancer cells ACHN, OS-RC-2, and 786-O, with IC_50_ values ranging from 27.0 to 57.8 µmol/L (Luo et al. 2019a). Preussin, isolated from the fungus *Aspergillus candidus* KUFA 0062, showed cell death by caspase-3 immunostaining. It exerted cytotoxic and anti-proliferative effects in breast cancer cell lines MCF7, SKBR3, and MDA-MB-231 (Malhão et al. 2019).

The two compounds, physcion and 2-(20,3-epoxy-10,30,50-heptatrienyl)-6-hydroxy-5-(3-methyl-2-butenyl) benzaldehyde (**26**), had selective cytotoxicity against glucose-deprived human pancreatic carcinoma PANC-1 cells, with IC_50_ values of 6.0 µmol/L and 1.7 µmol/L. These compounds were obtained from the fungus *Aspergillus* sp. (Abdel-Naime et al. 2020).

The extract of *Aspergillus oryzae* had cytotoxic properties against breast cancer cell T47D with IC_50_ value of 743.42 µg/mL (Handayani et al. 2020). Four *Aspergillus* sp., isolated from the three sponges *Tedania anhelans*, *Myxilla arenaria*, and *Callyspongia fibrosa*, had cytotoxic properties against NCI-H460 lung cancer cells (Lekshmi et al. 2020). The mycelium extract of an *Aspergillus* sp. isolated from a sponge collected from Kepulauan Seribu Marine National Park showed strong cytotoxicity against T47D cells with IC_50_ of 28.3 µg/mL as compared to the broth extract (IC_50_ = 645 µg/mL) (Nursid et al. 2020). The fungi *Aspergillus versicolor* MN859970 and *Aspergillus sydowii* MN8599 had cytotoxic properties against T47D cancer, with IC_50_ values of 1,760.98 ppm and 456.75 ppm (Sandrawati et al. 2020). Wang et al. (2020a) isolated *Aspergillus candidus* OUCMDZ-1051 from an unknown sponge. The compound 4-*O*-methylcandidusin A (**27**) had cytotoxic properties against 26 human cancer cell lines. It selectively inhibited MDA-MB-468, BT474, and A431 cancer cells with IC_50_ values of 1.84, 6.05, and 0.98 µmol/L.

**Figure 2.**
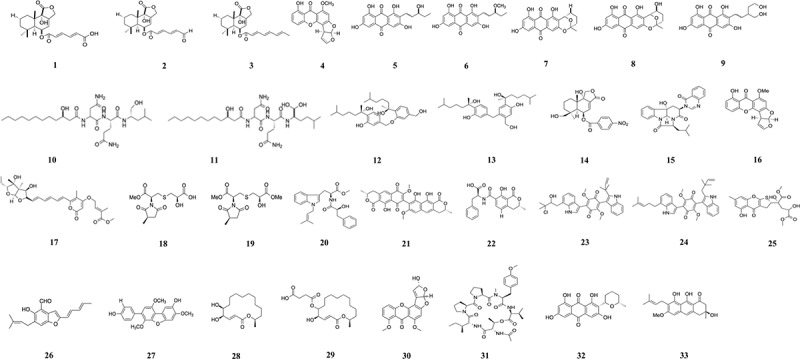
Compounds isolated from *Aspergillus* species associated with marine sponges.

The fungus *Aspergillus flocculosus* 01NT-1.1.5 was found to produce (5*R*,6*S*,16*R*,3*E*)-5,6-dihydroxy-16-methyloxacyclohexadec-3-en-2-one (**28**) and bekeleylactone E (**29**). These compounds showed cytotoxicity against the six cancer cells PC-3, HCT-15, MDA-MB-231, ACHN, NCI-H23, and NUGC-3 with GI_50_ values 1.1–3.6 µmol/L (Anh et al. 2021). *Aspergillus nomius* NC06 was isolated from the Indonesian sponge *Neopetrosia chaliniformis* and produced four compounds that were able to induce apoptosis and cell death in HT29 colon cancer cells. The compounds were new oxisterigmatocystins J (**30**) and K, L, and the known compounds aspergillicin A (**31**) (Artasasta et al. 2021). The IC_50_ values obtained in this study ranged from 1.63 to 988.05 µmol/L. The extract of the marine fungi *Aspergillus unguis*, *Aspergillus flavus*, *Aspergillus austroafricanus* had cytotoxic properties against T47D cells, with IC_50_ ranging from 365 to 1,500 µg/mL (Handayani et al. 2021). The sponge endophyte, *Aspergillus* sp. media-free and whole extract, induced apoptosis in HeLa cells with IC_50_ of 158.13 and 283.95 µg/mL, respectively (Pramana et al. 2022). *Aspergillus carneus* produced the five compounds arugosin C, averufin, averufanin (**32**), nidurifin, and versicolorin C. These compounds were tested on the breast, ovarian, and glioblastoma cancer cell lines, but it was averufanin (**32**) that had the lowest IC_50_ (0.28 ± 0.1 µmol/L) against MCF-7 cells (Demirel et al. 2023). The compound vismione E (**33**), isolated from the *Aspergillus* sp. 1901NT-1.2.2, had strong cytotoxicity against MCF-7 cells, with an IC_50_ of 9.0 ± 0.4 µmol/L (Girich et al. 2023). Figure 2 shows the most active compounds isolated from sponge-associated *Aspergillus*.

### 3.2. *Compounds from* Aspergillus *associated with algae*

The endophyte, *Aspergillus flavus*, was isolated from the marine algae *Enteromorpha tubulosa*. It produced the new diketopiperazine alkaloid, L-7,9-dihydroxy-8-methoxyphenylalanine, which showed cytotoxicity against HL-60 cell line with IC_50_ value of 36.5 µg/mL (Lin et al. 2007). The endophytic fungus *Aspergillus flavus* produced two compounds iso-α-cyclopiazonic acid (iso-α-CPA) (**34**) and α-CPA with cytotoxic properties against HL-60, MOLT-4, A-549, and BEL-7402. The IC_50_ values obtained with the two compounds ranged from 2.4 to >100 µmol/L (Table 2) (Lin et al. 2009). The fungus *Aspergillus versicolor*, obtained from the green algae *Halimeda opuntia*, produced the compound isorhodoptilometrin-1-methyl ether; 3-(2-hydroxy-propyl)-1-methyl ether-6,8-dihydroxy-9,10-anthra-quinone with mild solid tumour selectivity against HepG2 as compared to the normal cells (Hawas et al. 2012). *Aspergillus wentii* EN-48, obtained from the brown algae *Sargassum*, produced the compounds asperolide A, B, and wentilactone A, B. These compounds showed weak cytotoxicity against the cancer cells HeLa, HepG2, MCF-7, NCI-H460, SMMC-7721, and SW1990. Wentilactone B was the most potent with IC_50_ = 17 µmol/L (Sun et al. 2012). When tested against the cancer cells SMMC-7721, wentilactone B displayed IC_50_ of 31 µmol/L after 24 h and 19 µmol/L after 48 h (Zhang et al. 2012, 2013). The algal endophyte, *Aspergillus sydowii*, had cytotoxic properties against T47D cells with IC_50_ of 59.6 µg/mL (Fajarningsih et al. 2013). The anticancer effects on the breast cancer MCF-7 cells of the compound fumigaclavine C, isolated from the algal endophyte *Aspergillus fumigatus,* were investigated by Li et al. (2013). Ten cancer cell lines were used by Fang et al. (2014) in order to test the cytotoxicity of 6β,9α-dihydroxy-14-p-nitrobenzoylcinnamolide (**35**) and insulicolide A (**36**) isolated from *Aspergillus ochraceus* Jcma1F17. These showed significant cytotoxicity with IC_50_ values of 1.95–6.35 µmol/L. Isosclerone, isolated from the green algal endophyte *Aspergillus fumigatus*, had cytotoxic properties against MCF-7 cells with IC_50_ values of 63.92 and 39.77 µmol/L after treatment for 24 h and 36 h, respectively (Li et al. 2014b).

**Table 2.** Compounds isolated from *Aspergillus* associated with algae.

Algae species	Region/country	Media	Fungi	Compound	Cancer cell line	Activity (IC_50_)	Reference
Marine algae *Enteromorpha tubulosa*	Putian Pinghai, China	Glucose, yeast extract, maltose, mannitol, MSG, corn plasm	*Aspergillus flavus* C-F-3	Iso-α-cyclopiazonic acid (iso-α-CPA) (**34**), α-CPA	HL-60, MOLT-4, A-549, BEL-7402	2.4 to >100 µmol/L	Lin et al. (2009)
*Coelarthrum* sp.	Paracel Islands, South China Sea	Maltose, malt extract, yeast extract, petone, potassium dihydrogen phosphate	*Aspergillus ochraceus* Jcma1F17	6β,9α-dihydroxy-14-p-nitrobenzoylcinnamolide (**35**), insulicolide A (**36**)	H1975, U937, K562, BGC-823, Molt-4, MCF-7, A549, Hela, HL60, Huh-7	1.95–6.35 µmol/L Positive control (trichostatin A): 0.03–0.16 µmol/L	Fang et al. (2014)
Red alga *Ahnfeltiopsis flabelliformis*	Dalian intertidal zone	Potato juice, sucrose	*Aspergillus unguis* DLEP2008001	Depsidone aspergillusidone C (**37**), aspergillusether A	A549	0.5 µg/mL, 28.6 µg/mL Adriamycin: 0.008 µg/mL	Zhang et al. (2014)
Marine alga	Qingdao, China	Mannitol, glucose, peptone, yeast extract	*Aspergillus tennesseensis* strain OUCMB I 140430	Dihydrobenzofuran derivative 3-(2-(1-hydroxy-1-methyl-ethyl)-6-methyl-2,3-dihydrobenzofuran-4-yloxy)-5-methylphenol (**38**)	THP-1	7.0 µg/mL	Li et al. (2018c)
*Coelarthrum* sp.	South China Sea	MB	*Aspergillus ochraceus* Jcma1F17	14-*O*-acetylinsulicolide A (**39**), insulicolide A, B, C, 6β,9α-dihydroxy-14-p-nitrobenzoylcinnamolide, 9-deoxy insulicolide A	ACHN, OS-RC-2, 786-O	0.89–30 µmol/L Positive control (sorafenib): 3.4–7.0 µmol/L	Tan et al. (2018)
*Padina* sp.	Son Tra peninsula, Da Nang, Vietnam	Rice medium	*Aspergillus flocculosus* 168ST-16.1	14,15-dehydro-6-*epi*-ophiobolin K (**40**), 14,15-dehydro-ophiobolin K (**41**), 14,15-dehydro-6-*epi*-ophiobolin G (**42**), 14,15-dehydro-ophiobolin G (**43**), 14,15-dehydro-(Z)-14-ophiobolin G (**44**), 6-*epi*-ophiobolin C (**45**), ophiobolin C (**46**), 6-*epi*-ophiobolin N (**47**), ophiobolin N (**48**)	HCT-15, NUGC-3, NCI-H23, ACHN, PC-3, MDA-MB-231	0.14–2.01 µmol/L Adriamycin: 0.13–0.16 µmol/L	Choi et al. (2019)
*Kappaphycus alvarezii*	Vietnam	–	*Aspergillus mcronesiensis*	Aspermicrones B (**49**)	HepG2, LU-1, Vero	9.9 µmol/L against HepG2 Doxorubicin: 0.53, 0.57 µmol/L	Luyen et al. (2019)
*Ulva lactuca*	Northeast Taiwan of China	Rice medium	*Aspergillus giganteus* NTU967	Aspergilsmin C (**50**), patulin (**51**)	SK-Hep-1, PC-3	2.70 ± 0.1 to 7.3 ± 0.3 µmol/L Paclitaxel: 0.011 ± 0.002, 0.013 ± 0.002 µmol/L	Chen et al. (2020)
*Enteromorpha* sp.	Konkan coast	PDB	*Aspergillus unguis* AG1.1	*trans*-9-octadecenoic acid, hexadecanoic acid, octadecanoic acid, prosta-5,13-dien-1-oic acid, *cis*-4,7,10,13,16,19-docosahexaenoic acid, oleic acid, 4-(4-hydroxy-3,5-dimethoxy-phenyl)-3,4-dihydro-1 H-benzo[h]quinolin-2-one, 1-hydroxy-3,5-dimethoxy-2-prenylxanthone, 1,6-dihydroxy-3-methoxy-2-prenylxanthone, diethyl phthalate, asperxanthone (**52**), 3-butylidene-7-hydroxyphthalide (**53**), 01-ethyl 04-(2-hydroxyethyl) benzene-1,4-dicarboxylate	HeLa, MCF-7, A431, COLO 205, HEK293	13.46 ± 0.89 to >100 µg/mL	Sajna et al. (2020)

Gliotoxin was isolated from the fungus *Aspergillus* sp. It was able to inhibit the growth of HeLa cells by 73% at 36 h and SW1353 cells (59%) at 48 h (Nguyen et al. 2014). The broth extract fraction (F8) of the marine algae endophyte *Aspergillus terreus* showed cytotoxic properties against HepG2 cells with GI_50_ < 10 µg/mL (Suja et al. 2014). A depsidone, aspergillusidone C (**37**), isolated from the red seaweed fungus *A. unguis* DLEP200800, showed strong cytotoxicity against A549 cells. An IC_50_ of 0.5 µg/mL was obtained by Zhang et al. (2014). *Aspergillus niger* SCSIO Jcsw6F30 was found to produce aurasperone F which had cytotoxic activities against HeLa, MCF-7, Molt-4, Huh-7, and H1975 at concentration of 30 µmol/L (Fang et al. 2016). Gliotoxin, obtained from the fungus *Aspergillus fumigatus*, caused apoptosis in HT1080 human fibrosarcoma cells (Kim and Park 2016).

Demethoxyfumitremorgin C, obtained from the algal endophyte *Aspergillus fumigatus*, had anti-proliferative effects on PC3 human prostate cancer cells (Kim et al. 2017). The dihydrobenzofuran derivative 3-(2-(1-hydroxy-1-methyl-ethyl)-6-methyl-2,3-dihydrobenzofuran-4-yloxy)-5-methylphenol (**38**) isolated from the algal endophyte *Aspergillus tennesseensis* displayed considerable cytotoxicity against THP-1 cell line, with IC_50_ value of 7.0 µg/mL (Li et al. 2018c). The marine fungi *Aspergillus ochraceus* Jcma1F17 produced the compounds 14-*O*-acetylinsulicolide A (**39**), insulicolide A, B, C, 6β,9α-dihydroxy-14-p-nitrobenzoylcinnamolide, 9-deoxy insulicolide A, which had cytotoxic properties against ACHN, OS-RC-2, and 786-O. Insulicolide A had the strongest cytotoxicity with IC_50_ of 1.5, 1.5, and 0.89 µmol/L against ACHN, OS-RC-2, and 786-O cells, respectively (Tan et al. 2018). Five new sesterterpenes and four known ophiobolins were obtained from the marine algal endophyte *Aspergillus flocculosus* 168ST-16.1. These include 14,15-dehydro-6-*epi*-ophiobolin K; 14,15-dehydro-ophiobolin K; 14,15-dehydro-6-*epi*-ophiobolin G; 14,15-dehydro-ophiobolin G; 14,15-dehydro-(*Z*)-14-ophiobolin G; 6-*epi*-ophiobolin C; ophiobolin C; 6-*epi*-ophiobolin N; and ophiobolin N (**40–48**). These compounds were cytotoxic against the six cancer cell lines HCY-15, NUGC-3, NCI-H23, ACHN, PC-3, and MDA-MB-231. 14,15-dehydro-6-*epi*-ophiobolin K (**40**) displayed the strongest cytotoxicity against the HCT-15, NUGC-3, and MDA-MB-231 cell lines with GI_50_ values of 0.21, 0.19, and 0.14 µmol/L, respectively (Choi et al. 2019). Luyen et al. (2019) isolated the compound aspermicrone B (**49**) from the algal endophyte *Aspergillus micronesiensis*. This compound had selective cytotoxicity against HepG2 (IC_50_ = 9.9 µmol/L). *Aspergillus* sp. XNM-4, isolated from *Leathesia nana*, produced the cytotoxic compounds asperpyrone A, B, and aurasperone F against the cancer cells PANC-1, A549, MDA-MB-231, Caco-2, and SK-OV-3. Asperpyrone A had stronger cytotoxicity against PANC-1 with IC_50_ value of 8.25 ± 2.20 µmol/L (Xu et al. 2018).

*Aspergillus giganteus* NTU967 was isolated from the green algae *Ulva lactuca*. It produced two cytotoxic compounds, aspergilsmin C (**50**) and patulin (**51**), which displayed cytotoxic properties against SK-Hep-1 and PC-3 cells, with IC_50_ ranging from 2.70 ± 0.1 to 7.3 ± 0.3 µmol/L (Chen et al. 2020). The marine red algae *Laurencia obtuse* contained the endophytic fungus *Aspergillus niger* ASSB4. The latter produced the compounds RF-3192C, dimeric coumarin orlandin, fonsecin B, TMC-256A1, cyclo-(Leu-Ala), and cerebroside A. These had varying cytotoxic properties against the five cancer cells (IC_50_: 9.34–48.7 µg/mL) used in the study by Mahmoud et al. (2020). The *Enteromorpha* sp. endophyte *A. unguis* AG1.1 was found to produce the five metabolites 4-(4-hydroxy-3,5-dimethoxy-phenyl)-3,4-dihydro-1H-benzo[h]quinolin-2-one, 1-hydroxy-3,5-dimethoxy-2-prenylxanthone, 1,6-dihydroxy-3-methoxy-2-prenylxanthone, asperxanthone (**52**), 3-butylidene-7-hydroxyphthalide (**53**). These had IC_50_ values ranging from 13.46 ± 0.89 to 19.97 ± 0.21 µg/mL against the cancer cells HeLa, MCF-7, A-431, and COLO 205 (Sajna et al. 2020).

**Figure 3.**
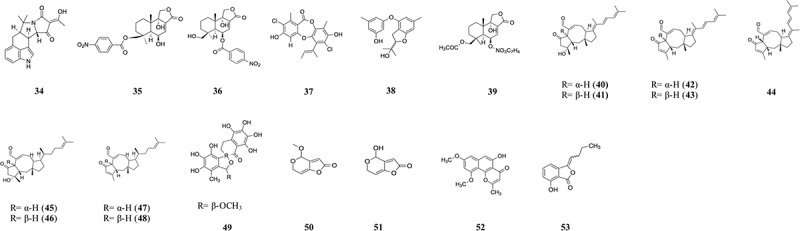
Compounds isolated from *Aspergillus* associated with algae.

Sahoo et al. (2021) recovered the algal endophytes *Aspergillus amoenus*, *A. tubingensis*, *A. terreus*, *A. ochraceopetaliformis*, *A. amstelodami*, *A. niger*, and *A. tamari*. Their crude extracts had cytotoxic properties against A431 and HeLa cells, with IC_50_ values 42.17–165.53 µg/mL. The *Aspergillus* sp. isolated from the marine seaweed *S. muticum* was able to inhibit the growth of six cancer cell lines, with HeLa cells being more susceptible (IC_50_ = 24 ± 2 µg/mL), followed by MCF-7 cells (IC_50_ = 32.0 ± 2.3 µg/mL) and HepG2 cells (IC_50_ = 33 ± 2.3 µg/mL). It was non-toxic to the human embryonic kidney non-cancerous HEK 293T cells (Taritla et al. 2021). Figure 3 shows the most active compounds derived from algae associated *Aspergillus*.

### 3.3. *Compounds from* Aspergillus *obtained from sediments*

#### 3.3.1. Marine sediment-derived fungi

**Figure 4.**
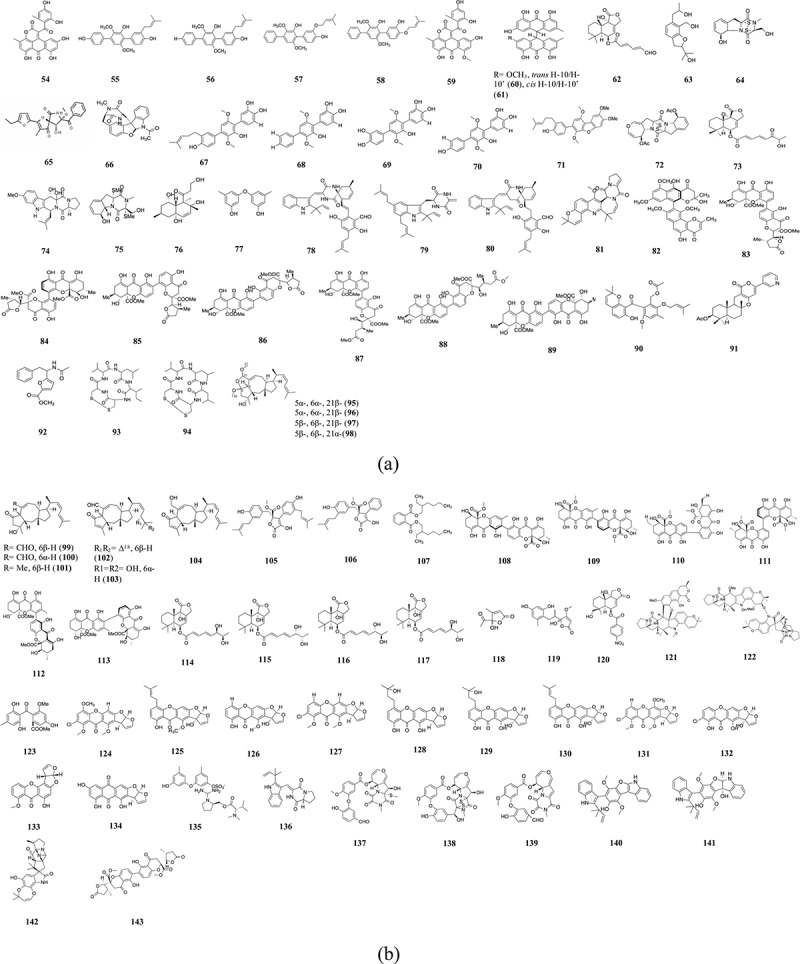
Compounds from *Aspergillus* obtained from sediments. (a) Compounds (**54–98**). (b) Compounds (**99–143**).

Aspergiolide A (**54**) (Figure 4a), isolated from *A. glaucus*, was cytotoxic against A-549, HL-60, BEL-7402, and P388 cell lines, with IC_50_ values of 0.13, 0.28, 7.5, and 35.0 µmol/L, respectively (Table 3) (Du et al. 2007). 2,3’-dimethylosoate and monomethylsulochrin were isolated from the fungus *Aspergillus* sp. B-F-2. These two compounds showed cytotoxicity against K562 cell line with IC_50_ values of 76.5 µmol/L and >100 µmol/L (Liu et al. 2006).

**Table 3.** Compounds from *Aspergillus* obtained from sediments.

Source	Region/country	Media	Fungi	Compound	Cancer cell line	Activity (IC_50_)	Reference
Sediments around mangrove roots	Fujian Province, China	Mannitol, maltose, glucose, MSG, yeast extract, corn steep liquor	*Aspergillus glaucus*	Aspergiolide A (**54**)	A-549, HL-60, BEL-7402, P388	0.13, 0.28, 7.5, 35.0 µmol/L	Du et al. (2007)
Sediment collected from a depth of 50 m	Gokasyo Gulf, Mie Prefecture, Japan	Malt extract, glucose, peptone	*Aspergillus candidus* IF10	Prenylterphenyllin (**55**), 4’-deoxyprenylterphenyllin (**56**), 4’-deoxyisoterprenin (**57**), 4’-deoxyterprenin (**58**)	KB3-1	8.5, 3.0, 2.5, 4.5 µg/mL	Wei et al. (2007)
Sediment surrounding mangrove roots	Fujian Province, China	Mannitol, maltose, glucose, MSG, yeast extract, corn steep liquor	*Aspergillus glaucus*	Aspergiolide B (**59**), (*trans*)-, (*cis*)-emodin-physcion bianthrone (**60**, **61**)	HL-60, A549	0.24–44.0 µmol/L	Du et al. (2008)
Rhizosphere soil of the mangrove plant *Bruguiera gymnorrhiza*	Wenchang, Hainan Province of China	Mannitol, glucose, maltose, yeast extract, MSG, corn steep liquor	*Aspergillus ustus* 094102	Ustusorane E (**63**), ustusolates A, C, E (**62**)	A549, HL-60	0.13 to >100 µmol/L Etoposide: 0.63, 0.042 µmol/L	Lu et al. (2009)
Sediments	Jiaozhou Bay of Qingdao, China	Mannitol, glucose, maltose, beef extract, MSG, corn steep liquor	*Aspergillus fumigatus* Fres	Gliotoxin (**64**), didehydro-bisdethiobis (methylthio) gliotoxin	tsFT210	0.15, 89.8 µg/mL	Zhao et al. (2009)
Sediments	Jiaozhou Bay, China	Mannitol, glucose, maltose, MSG, corn syrup	*Aspergillus sydowi* D2-6	Azaspirofurans A (**65**)	A549	10 µmol/L	Ren et al. (2010)
Sediments	Hawaii	Czapek-Dox liquid medium	*Aspergillus insulicola*	Azonazine (**66**), diazonamide A, insulicolide A	Murine colon 38, LNCaP, CEM, HCT-116	<15 ng/mL against HCT-116	Wu et al. (2010)
Root soil of mangrove plant *Acrostichum aureum*	–	Mannitol, maltose, glucose, MSG, yeast extract, corn steep liquor	*Aspergillus taichungensis* ZHN-7-07	Prenylterphenyllins A (**67**)-C, prenylcandidusin A-C, prenylterphenyllin, 4”-dehydro-3-hydroxyterphenyllin (**68**), prenylcandidusins B, 3-hydroxyterphenyllin, terphenyllin (**70**), 3,3-dihydroxyterphenyllin (**69**), prenylcandidusin A, B (**71**), C	HL-60, A-549, P388	1.53 to >100 µmol/L	Cai et al. (2011a)
Marine sediment	Ulleung Basin, East Sea of Korea	Czapek-Dox	*Aspergillus* sp. KMD 901	Acetylaranotin, acetylapoaranotin (**72**), deoxyapoaranotin	HCT116, AGS, A549, MCF-7, HepG2, 3T3-L1	2 ± 1.08 to >200 µmol/L	Choi et al. (2011)
Mangrove rhizosphere soil	Guangxi Province, China	Mannitol, glucose, maltose, MSG, Yeast extract, corn steep liquor	*Aspergillus ustus*	(6-strobilactone-B) ester of (*E,E*)-6-carbonyl- 7-hydroxy-2,4-octadienoic acid (**73**)	P388	8.7 µmol/L	Zhou et al. (2011)
Sea mud	Yingkou, China	Mannitol, glucose, maltose, MSG, yeast extract, corn steep liquor	*Aspergillus fumigatus* YK-7	Prenylcyclotryprostatin B, 20-hydroxycyclotryprostatin B, 9- hydroxyfumitremorgin C, 6-hydroxytryprostatin B, spirogliotoxin, cyclotryprostatins A and B, fumitremorgin B, fumitremorgin C, 12,13-dihydroxyfumitremorgin C (**74**), verruculogen, gliotoxin, bisdethiobis(methylthio)gliotoxin (**75**), didehydrobisdethiobis(methylthio)gliotoxin	U937, PC-3	0.20 ± 0.03 to >100 µmol/L Doxorubicin hydrochloride: 0.021 ± 0.002 µmol/L, 0.73 ± 0.044 µmol/L	Wang et al. (2012)
Sediments	Russia	Rice medium	*A. sulphureus* KMM 4640	Decumbenone C (**76**), decumbenones A, B, diorcinol (**77**), brevianamide F	SK-MEL-28, SK-MEL-5 human melanoma, HCT 116	0.9 to >160 µmol/L	Zhuravleva et al. (2012)
Mangrove rhizosphere soil	Fujian Province, China	Glucose, maltose, MSG, beef extract	*Aspergillus effuses* H1-1	Dihydroneochinulin B (**79**), cryptoechinuline D (**78**), (+)-cryptoechinuline D, (-)-cryptoechinuline D (**80**), didehydroechinulin B	P388, HL-60, BEL-7402, A-549	1.43 to >100 µmol/L Doxorubicin: 0.05–0.33 µmol/L	Gao et al. (2013b)
Sediments (depth 35 m)	South China Sea	Maltose, mannitol, glucose, peptone, yeast extract-potato	*Aspergillus versicolor* HDN08-60	Versicamide H (**81**)	HeLa, HCT-116, HL-60, K562	8.7–22.4 µmol/L	Peng et al. (2014)
Mud	Huludao coastline, Liaoning Province, China	Mannitol, glucose, yeast extract, peptone, corn syrup	*Aspergillus niger* 2HL-M-8	Aurasperoe H, fonsecinones C (**82**)	A549, MGC-803, HL-60	0.8–67.1 µmol/L	Li et al. (2015)
Soil around a mangrove	Guangzhou, China	Maltose, mannitol, glucose, MSG, yeast extract, corn steep liquor	*Aspergillus versicolor* HDN1009	Versixanthone A–F (**83–88**) secalonic acid D (**89**)	HL-60, K562, A549, H1975, 803, H0-8910, HCT-116	0.7 to >50 µmol/L Dox: 0.02–0.8 µmol/L	Wu et al. (2015)
Rhizosphere soil of mangrove *Thespesia populnea*	Guangxi Province, China	Potato broth, maltose, peptone, mannitol, yeast extract	*Aspergillus versicolor* HDN11-84	Versicones E-H, arugosin K (**90**)	K562, Hela, NB_4_, HL-60, HCT-116	9.2 to >50 µmol/L Doxorubicin: 0.1–0.6 µmol/L	Li et al. (2016a)
Sea mud	Yingkou, China	Maltose, mannitol, glucose, MSG, yeast extract, corn steep liquor	*Aspergillus fumigatus* YK-7	β-5,8,11-trihydroxybergamot-9-ene, β-trans-2β,5,15-trihydroxybergamot-10-ene, alismol, pyripyropene E (**91**), helvolic acid	U937, PC3	4.2 ± 0.3 to >100 µmol/L Doxorubicin hydrochloride: 0.0021 ± 0.002 µmol/L, 0.73 ± 0.04 µmol/L	Wang et al. (2015)
Marine sediments	Northeastern coast of Brazil	Malt peptone dextrose broth	*Aspergillus niger* BRF-074	Furan ester derivative (**92**), malformin A1 (**93**), malformin C (**94**)	HCT-116	2.9 µg/mL	Uchoa et al. (2017)
Rhizosphere soil of *Bruguiera gymnorrhiza*	-	Maltose, mannitol, glucose, MSG, yeast extract, corn steep liquor	*Aspergillus ustus* 094102	Ophiobolin G, Q (**102, 103**), ophiobolin Z; 21-*epi*-ophiobolin Z; 21-*epi*-ophiobolin O; ophiobolin O (**95–98**), ophiobolin K; 6-*epi* ophiobolin K; 21-deoxyophiobolin K (**99–101**), 6-*epi*-ophiobolin G (**104**), ophiobolin H, K, P, Q, U; 21-dehydroophiobolin U; 21,21-*O*-dihydro-6-*epi*-ophiobolin G; 5,6-di-*epi*-ophiobolin H	G3K, MCF-7, MD-MBA-231, MCF/Adr, A549, HL-60, MCF-10A	0.6 to >50 µmol/L Adriamycin: 0.02–45.0 µmol/L	Zhu et al. (2017)
Sea deposit	Fengxian Bay, Shanghai, China	Rice medium	*Aspergillus terreus*	(+)-3’,3’-di-(di-methylallyl)-butyrolactone II (**105**), versicolactone B (**106**)	PANC-1, HCC1806, HepG2, BEAS-2B, HT-29	5.3, 9.4 µmol/L	Qi et al. (2018)
Sediment	River Nile, Egypt	Sabouraud dextrose broth	*Aspergillus awamori*	Di-(2-ethylhexyl) phthalate (**107**)	MCF7, HepG2, Hela, HCT116	6.525–66.607 µg/mL	Lofty et al. (2018)
Soil around a mangrove	Guangzhou, China	Maltose, mannitol, glucose, MSG, corn steep liquor	*Aspergillus versicolor* HDN1009	Versixanthones G, H, L, M (**108–111**)	HL-60, K562, A549, H1975, MGC803, HEK 293, HO-8910, HCT-116	0.4 to >50.0 µmol/L	Wu et al. (2018)
Soil around a mangrove	Guangzhou, China	Maltose, mannitol, glucose, MSG, corn steep liquor	*Aspergillus versicolor* HDN1009	5-*epi*-asperdichrome, versixanthones N, O (**112, 113**)	HL-60, K562, H1975, MGC803, HO-8910, A549	1.7 to >30 µmol/L Dox: 0.02–0.8 µmol/L	Yu et al. (2018)
Sediment	Bohai Sea	Rice medium	*Aspergillus flavus* CF13-11	Asperiene A–D (**114–117**)	HeLa, MCF-7, MGC-803, A549, GES-1	1.4–8.3 µmol/L	Liu et al. (2019)
Saline soil	Bohai Bay, Zhanhua	Potato, glucose, maltose, mannitol, yeast extract	*Aspergillus sclerotiorum* JH42	Aspersclerolide A, C (**118, 119**)	HL60, A549, HL-7702	8.9–22.8 µmol/L Dox: 0.85–8.3 µmol/L	Ma et al. (2019)
Sediment	Nha Trang Bay, South China Sea, Vietnam	Rice, yeast extract	*Aspergillus flocculosus*	6β,9α,14-trihydroxycinnamolide, aspertetranones D, A, 7α,14-dihydroxy-6β-p-nitrobenzoylconfertifolin, insulicolide A (**120**), aspilactonol F, G, 12-*epi*-aspertetranone D, 6β,7β,14-trihydroxyconfertifolin, dihydroaspirone	22Rv1, MCF-7, Neuro-2a	3.0 to >100 µmol/L Docetaxel: 0.02 µmol/L	Yurchenko et al. (2019)
Soil	Waikiki beach, Oahu, Hawaii	Mannitol, glucose, MSG, yeast extract	*Aspergillu*s sp. FM 242	Waikikiamides A, C (**121, 122**)	HT1080, PC3, Jurkat, A2780S	0.56–1.86 µmol/L	Wang et al. (2020b)
Sediment	Canyon Dahab, Red Sea, Egypt	Rice medium	*Aspergillus falconensis*	Sulochrin (**123**)	L5178Y	5.1 µmol/L	El-Kashef et al. (2020)
Marine sediments	Jeju-do Korea	Yeast extract, malt extract, mannitol, rice	*Aspergillus ochraceopetaliformis*	Ochraceopetalin (**135**), 1-(sulfooxy)-diorcinol, ochraceopetaguanidine, diorcinol	A549, K562	6.8–25 µmol/L	Park et al. (2021)
Sea mud	Coast of Bohai, China	Rice, modified Martin medium	*Aspergillus versicolor* HBU-7	(+)-brevianamide V (**136**)	HGC-27	4.54 µmol/L Cisplatin: 8.63 µmol/L	Li et al. (2022a)

The fungus *Aspergillus variecolor* B-17 was obtained from the sediments in Mongolian Jilantai Salt Field, China. Three new alkaloids, variecolortides A–C, were isolated from this fungus and these had cytotoxic activities against K-562 cells with IC_50_ values of 61, 69, and 71 µmol/L (Wang et al. 2007). Prenylterphenyllin (**55**), 4’-deoxyprenylterphenyllin (**56**), 4’-deoxyisoterprenin (**57**), and 4’-deoxyterprenin (**58**) were isolated from *Aspergillus candidus* IF10. These four compounds were cytotoxic against KB3–1 cells with IC_50_ of 8.5, 3.0, 2.5, and 4.5 µg/mL, respectively (Wei et al. 2007). Carbonarones A and B were isolated from *Aspergillus carbonarius* WZ-4-11 and had moderate cytotoxicity against the human leukaemia K562 cells with IC_50_ values of 56.0 and 27.8 µg/mL, respectively (Zhang et al. 2007). Du et al. (2008) isolated *Aspergillus glaucus* which produced three compounds, aspergiolide B (**59**) and two new bianthrones (*trans*)-, (*cis*)-emodin-physcion bianthrone (**60**, **61**). These compounds showed cytotoxic properties against HL-60 and A549, with IC_50_ values ranging from 0.24 to 44.0 µmol/L. The marine-derived fungus, *Aspergillus ustus* 094102, was found to produce cytotoxic ustusorane E (**63**) and ustusolates A, C and ustusolate E (**62**). The IC_50_ values ranged from 0.13 to >100 µmol/L (Lu et al. 2009). The fungus *Aspergillus variecolor* B-17, obtained from sediments, produced the cytotoxic compound 2-hydroxydiplopterol. It showed cytotoxicity against K562 cells with IC_50_ of 22 µmol/L (Wang et al. 2009). Gliotoxin (**64**) and didehydro-bisdethiobis (methylthio) gliotoxin were obtained from *Aspergillus fumigatus* Fres. These showed cytotoxic properties against tsFT210 with IC_50_ 0.15 µg/mL and 89.8 µg/mL, respectively (Zhao et al. 2009).

Azaspirofurans A (**65**) isolated from the sediment-derived fungus *Aspergillus sydowi* D2-6, displayed cytotoxic activity against A549 cells, with IC_50_ value of 10 µmol/L (Ren et al. 2010). Wu et al. (2010) isolated azonazine (**66**), diazonamide A, and insulicolide A from the fungus *Aspergillus insulicola*. These compounds had cytotoxic properties against Murine colon 38, LNCaP, and HCT-11 cancer cells. N-acetyltyramine, isolated from *Aspergillus fumigatus* Fres., was cytotoxic against A375 and K562 cells. An IC_50_ value of 17.4 µmol/L was obtained against K562 cells (Zhao et al. 2010). The compounds prenylterphenyllin A (**67**) and prenylterphenyllin displayed cytotoxic properties against the three cell lines HL-60, A-549, and P388 (IC_50_: 1.53–10.90 µmol/L), while 4”-dehydro-3-hydroxyterphenyllin (**68**) and prenylcandidusin B (**71**) had cytotoxic activities against *p*-388 cell line (IC_50_: 2.70 and 1.57 µmol/L). Three other compounds 3-hydroxyterphenyllin, terphenyllin (**70**), and 3,3-dihydroxyterphenyllin (**69**) also showed moderate cytotoxicity. These compounds were obtained from the root soil fungus *Aspergillus taichungensis* ZHN-7-07 (Cai et al. 2011a). Diketopiperazine disulphides were purified from the sediment-derived fungi *Aspergillus* sp. SF-5044. These had cytotoxic properties against the cancer cells HCT116, AGS, A549, MCF-7, and HepG2. Acetylapoaranotin (**72**) showed stronger cytotoxic activities against the first four cell lines with IC_50_ values of 2 ± 1.08 to 13.8 ± 1.59 µmol/L (Choi et al. 2011). Another compound, protuboxepin A, was obtained from the culture of *Aspergillus* sp. SF-5044. This compound had weak inhibitory activity against the cancer cells MDA-MB-231, Hep3B, 3Y1, K562, and HL-60 with IC_50_ values ranging from 75 to 250 µmol/L (Lee et al. 2011a). *Aspergillus protuberus* sp. 1 was isolated by Mathan et al. (2011) from marine sediments of the South India coastal belt. The mycelium n-butanol extract had IC_50_ value of 125 µg/mL against Hep2 cells. Butyrolactone I, obtained from the fungus *Aspergillus terreus* PT06-2, showed weak cytotoxicity against HL-60 with an IC_50_ value of 57.5 µmol/L (Wang et al. 2011). The n-butanol extract of the mycelium showed IC_50_ of 125 µg/mL against Hep2 cells. Another fungus, *Aspergillus ustus*, produced drimane sesquiterpenoids (**73**) which were cytotoxic to P388 cells with IC_50_ of 8.7 µmol/L (Zhou et al. 2011).

Gao et al. (2012) isolated *Aspergillus effuses* H1-1 from the mangrove rhizosphere soil and obtained two compounds, effusin A and dihydrocryptoechinulin D, which showed cytotoxic properties against P388, HL-60, BEL-7402, and A549 cells, with IC_50_ values ranging from 1.83 ± 0.21 to >100 µmol/L. *Aspergillus* sp. AF119 was found to produce the compounds p-terphenyl derivatives, 4-dehydroxy-3”-hydroxyl-terphenyllin, terphenyllin, 3-hydroxyterphyllin, 3,3’-dihydroxyterphyllin and candidusins A, B. These compounds were cytotoxic against the cell lines HeLa, HepG-2, and MDA-MB-435, with IC_50_ values ranging from 10.1 ± 0.8 to >100 µmol/L (Liu et al. 2012). Weak cytotoxic activity (IC_50_ > 10 µmol/L) on the cell lines HCT-8, Bel-7402, BGC-823, and A2780 were obtained by Shen et al. (2012). They tested the compounds 7″-hydroxybutyrolactone III, terretrione A–C, butyrolactone I, cyclo(Leu-Pro), cyclo(Val-Pro), cyclo(IlePro), cyclo(Phe-Pro) obtained from *Aspergillus terreus*. Wang et al. (2012) demonstrated the cytotoxic activity of the two prenylated indole diketopiperazines (**74**, **75**) and nine known compounds obtained from *Aspergillus fumigatus* YK-7 against U937 and PC-3 cells. The IC_50_ values obtained ranged from 0.20 ± 0.03 to >100 µmol/L. Decumbenone C (**76**), a new cytotoxic compound isolated from the marine fungus *Aspergillus sulphureus* KMM 4640, had IC_50_ values of 0.9 µmol/L against the human melanoma SK-MEL-5. The known compound diorcinol (**77**) also showed strong cytotoxicity (Zhuravleva et al. 2012). From the same isolate, *Aspergillus effuses* H1-1, Gao et al. (2013b) also obtained cryptoechinuline D (**78**), dihydroneochinulin B (**79**), (+)-cryptoechinuline D, (-)-cryptoechinuline D (**80**), and didehydroechinulin B. They tested these against more cell lines, namely P388, HL-60, BEL-7402, A-549, and noted the cytotoxic properties of the additional compounds, with IC_50_ values ranging from 1.43 to >100 µmol/L. According to Gao et al. (2013a), the fungus *Aspergillus versicolor* ZLN-60 produced cytotoxic diorcinol D, E which had cytotoxic properties against HeLa and K562 cells. The IC_50_ values obtained were from 31.5 to 48.9 µmol/L. Epi-deoxybrevianamide E and sterigmatocystin were isolated from the fungus *Aspergillus versicolor* KMM 4644. These compounds were cytotoxic to HeLa and HL-60 tumor cells, with IC_50_ values of 46.7–117.5 µmol/L (Sobolevskaya et al. 2013).

The fungus *Aspergillus versicolor* HDN08-60 was found to produce versicamide H (**81**) that was cytotoxic against HeLa, HCT-116, HL-60, and K562 cells, with IC_50_ of 8.7–22.4 µmol/L (Peng et al. 2014). Pseurotin A, D, diketopiperazines fumitremorgin C, and 12,13-dihydroxy-fumitremorgin C were isolated from the sediment-derived *Aspergillus* sp. BRF 030. These were in higher concentration after 21 days and were responsible for the cytotoxic properties of the extract against HCT-116 (IC_50_: 4.53–85 µmol/L) (Saraiva et al. 2014). A new naphthopyrone, aurasperone H, and fonsecinones C (**82**) were isolated from the marine fungus *Aspergillus niger* 2HL-M-8. These compounds displayed cytotoxic properties against A549, HL-60, and MGC-803, with IC_50_ values ranging from 0.8 to 67.1 µmol/L (Li et al. 2015). According to Wu et al. (2015), versixanthones A–F (**83–88**) and secalonic acid D (**89**), isolated from *Aspergillus versicolor* HDN1009, had cytotoxic properties against the seven cancer cell lines HL-60, K562, A549, H1975, 803, H0-8910, and HCT-116. The IC_50_ values ranged between 0.7 to >50 µmol/L. Only versixanthone E was able to inhibit the activity of topoisomerase I.

Versicone G and arugosin K (**90**), obtained from the rhizosphere soil fungus *Aspergillus versicolor* HDN11-84, had cytotoxicity against NB4, HL-60, and Hela cells with IC_50_ ranging from 9.2 to 21.7 µmol/L (Li et al. 2016a). The coastal saline soil fungus *Aspergillus fumigatus* produced the sesquiterpenoid derivative, aspergiketone. This compound showed cytotoxicity against HL-60 and A549 with IC_50_ values of 12.4 and 22.1 µmol/L, respectively (Liu et al. 2016). The medium extract of the fungus *Aspergillus flavus* had cell inhibitory effects on the human breast adenocarcinoma cell MCF7 with IC_50_ of 29.03 µg/mL (Samuel and Sudarmani 2016). Another marine fungus, *Aspergillus fumigatus* YK-7 was found to produce the metabolites *E*-β-*trans*-5,8,11-trihydroxybergamot-9-ene and terpenoid pyripyropene E (**91**) which were cytotoxic to U937 and PC3 cells. The IC_50_ values obtained ranged from 4.2 ± 0.3 to >100 µmol/L (Wang et al. 2015). Marine mangrove sediments were collected from Pichavaram, Tamil Nadu, and the fungus *Aspergillus* sp. was isolated from it. Chitin and chitosan were extracted from this fungus and showed cytotoxicity against HeLa cells (Anandhi 2017). The two compounds, 3-methylpentyl-2,4-dichloroasterrate and butyl 2,4-dichloroasterrate, were produced by the wetland fungus *Aspergillus flavipes* PJ03-11. These compounds showed cytotoxic properties against the cancer cells HL-60, HCT-116, HT-29, and PC-3, with IC_50_ values ranging from 16.89 to 71.06 µmol/L (Liu et al. 2017a). The fungus *Aspergillus niger* BRF-074 produced a new furan ester derivative (**92–94**) which had cytotoxic properties against colon adenocarcinoma cell HCT-116, with IC_50_ value of 2.9 µg/mL (Uchoa et al. 2017). The 18 compounds, ophiobolin G, Q (**102, 103**), ophiobolin Z; 21-*epi*-ophiobolin Z; 21-*epi*-ophiobolin O; ophiobolin O (**95–98**), ophiobolin K; 6-*epi* ophiobolin K; 21-deoxyophiobolin K (**99–101**), 6-*epi*-ophiobolin G (**104**), ophiobolin H, K, P, Q, U; 21-dehydroophiobolin U; 21,21-*O*-dihydro-6-*epi*-ophiobolin G; and 5,6-di-*epi*-ophiobolin H, displayed cytotoxic activities against the cancer cells G3K, MCF-7, MD-MBA-231, MCF/Adr, A549, and HL-60 with IC_50_ values of 0.6 to >50 µmol/L (Figure 4b) (Zhu et al. 2017).

The marine fungi *Aspergillus terreus*, obtained from sea deposits, produced two compounds which showed promising anti-tumour activities against the pancreatic ductal adenocarcinoma (PANC-1). Compounds (+)-3’,3’-di-(di-methylallyl)-butyrolactone II (**105**) and versicolactone B (**106**) showed cytotoxic activities against PANC-1 cells, with IC_50_ values of 5.3 and 9.4 µmol/L, respectively (Qi et al. 2018). Lofty et al. (2018) isolated the compound di-(2-ethylhexyl) phthalate (**107**) from the River Nile fungus *Aspergillus awamori*. This compound showed cytotoxic properties against MCF7, HepG2, HeLa, and HCT116 cells with IC_50_ of 6.525, 26.73, 42.2958, and 66.607 µg/mL, respectively. The two compounds versicolorin B and nidurufin were isolated from the sediment-derived fungus *Aspergillus versicolor* A21-2-7. A weak cytotoxicity was observed on A549 with IC_50_ values of 25.97 and 25.60 µmol/L, respectively (Wu et al. 2018). The versixanthones G, H, L, and M (**108–111**), from the fungus *A. versicolor* HDN1009, showed differing cytotoxicity against eight cancer cell lines. The IC_50_ ranged from 0.5 µmol/L to >50 µmol/L against the cells HL-60, K562, A549, H1975, MGC803, HEK 293, HO-8910, and HCT-116 (Wu et al. 2018). The three compounds, 5-*epi*-asperdichrome, versixanthones N (**112**), O (**113**) exhibited strong cytotoxicity against the five cancer cell lines HL-60, K562, H1975, MGC803, and HO-8910 with IC_50_ values of 1.7 to >30 µmol/L. These compounds were derived from the soil fungus *Aspergillus versicolor* HDN1009 (Yu et al. 2018). Antonov et al. (2019) isolated *Aspergillus foetidus* KMM4694 from sediment. This fungus produced the compounds rubrofusarine B and fansecinones B which induced ROS production in the human drug-resistant prostate cancer 22Rv1 cells. Asperienes A–D (**114–117**) were isolated from the fungus *Aspergillus flavus* CF13-11. These four compounds were cytotoxic against the cancer cells HeLa, MCF-7, MGC-803, A549, and GES-1 with IC_50_ of 1.4–8.3 µmol/L (Liu et al. 2019). The soil-derived fungus, *Aspergillus sclerotiorum* JH42, displayed cytotoxic activities against HL60, A549, and HL-7702 cells, with IC_50_ values ranging from 8.9 to 22.8 µmol/L. It was the compounds γ-hydroxyl butenolide, aspersclerolide A (**118**), C (**119**), which were responsible for the bioactivity (Ma et al. 2019). The compounds 6β,9α,14-trihydroxycinnamolide and insulicolide A (**120**) were isolated from the sediment-derived fungus *Aspergillus flocculosus*. They displayed cytotoxic activities against the prostate cancer 22Rv1, human breast cancer MCF-7, and murine neuroblastoma Neuro-2a cells (IC_50_: 3.0 to >100 µmol/L) (Yurchenko et al. 2019).

Exopolysaccharides were isolated from the fungus *Aspergillus terreus* SEI. These showed cytotoxic activities against breast cancer and human skin fibroblast cell lines with IC_50_ >100 µg/mL and 47 µg/mL, respectively (Amer et al. 2020). The sediment fungus *Aspergillus falconensis* produced the two cytotoxic compounds falconensis A and R. These displayed NF-KB inhibitory activity against the breast cancer MDA-MB-231, and were cytotoxic at IC_50_ values 89.7 ± 9.1 µmol/L and 126.8 ± 5.4 µmol/L, respectively (El-Kashef et al. 2020). Waikikiamides A, C (**121, 122**) showed anti-proliferative activity against the cancer cell lines HT1080, PC3, Jurkat, and A2780S, with IC_50_ values ranging from 0.56 to 1.86 µmol/L. These compounds were isolated from the soil fungus *Aspergillus* sp. FM242 by Wang et al. (2020b).

The fungus *Aspergillus falconensis* was collected from the sediment of the Red Sea and the benzophenone derivative sulochrin (**123**) was isolated. This compound had cytotoxic activity against the mouse lymphoma cell-line L5178Y, with IC_50_ value of 5.1 µmol/L. Moreover, in the scratch wound assay, sulochrin (**123**) inhibited cell migration of breast cancer cells MDA-MB-231 at concentration of 70 µmol/L (El-Kashef et al. 2021). The marine soils inhabited the fungus *Aspergillus fumigatus* strain MF-1. It produced the compound 2,5-dioxocyclopentylamino-7-oxohepta-1,3,5-trienyl-2,5-dihydroxy-3-chlorophenyl-2,4,6-trimethyldeca2,4-dienamide which displayed cytotoxicity against HeLa cells with IC_50_ of 74.38 ± 0.31 µg/mL (Kalyani et al. 2021). Ochraceopetalin (**135**) had cytotoxic activities against the cancer cell lines A549 and K562, with IC_50_ values of 6.8 and 9.5 µmol/L, respectively. This compound was isolated from the sediment fungus *Aspergillus ochraceopetaliformis* (Park et al. 2021). *Aspergillus versicolor* HBU-7 produced the compound (+)-brevianamide V (**136**), which was cytotoxic against HGC-27 cell line. It had IC_50_ value of 4.54 µmol/L, which was stronger than the positive control cisplatin (IC_50_ 8.63 µmol/L) (Li et al. 2022a).

#### 3.3.2. Deep-sea sediment fungi

Fourteen compounds, namely aspiketolactonol, aspilactonols A–F, aspyronol, epiaspinonediol, (*S*)-2-(2′-hydroxyethyl)-4-methyl-γ-butyrolactone, dihydroaspyrone, aspinotriol A, aspinotriol B, and chaetoquadrin F, inhibited the human cancer cells K562, HL-60, HeLa, and BGC-823 cells. These compounds were obtained from the deep-sea sediment *Aspergillus* sp. 16-02-1 (Chen et al. 2014a). Fredimoses et al. (2015) isolated *Aspergillus westerdijkiae* SCSIO 05233 from deep-sea marine sediments. The compound, circumdatin G, showed inhibitory activities against the cells K562 and HL-60 with IC_50_ values of 25.8 and 44.9 µmol/L, respectively. Li et al. (2016c) isolated the fungus *Aspergillus wentii* SD-310 from a deep-sea sediment. The compound asperolide E had cytotoxic activities against HeLa, MCF-7, and NCI-H446 with IC_50_ values of 10.0, 11.0, and 16.0 µmol/L, respectively. The deep-sea sediment fungus *Aspergillus puniceus* SCSIOz021 had cytotoxic properties against Vero cells, with IC_50_ values ranging from 0.6 to 60 µmol/L. The compounds which were isolated were austocystin A, K, L, F, B, D, H (**124–130**), M, N, (1′*R*,2′*S*)-F02ZA-1593B2 (**131**), (1′*R*,2′*R*)-compound V (**132**), 8-*O*-methyldemethylsterigmatocystin (**133**), 8-*O*-methyldihydrodemethylsterigmatocystin, versicolorin B (**134**) (Figure 4b) (Liang et al. 2021). Niu et al. (2021) isolated the fungus *Aspergillus sydowii* MCCC 3A00324 from the deep-sea sediment. Two compounds, acremolin D and acremolin, were obtained from this fungus. These had cytotoxic properties against Hela-S3, K562 and A549, HepG2, and K562 cells, at concentration of 20 µmol/L. The fungus *Aspergillus puniceus* SCSIO z021 was isolated by Liu et al. (2022) in the deep-sea sediments. Among the different compounds, puniceusines D showed moderate cytotoxicity against human lung adenocarcinoma cell-line H1975 with IC_50_ value of 11.0 µmol/L. *Aspergillus nidulans* SD-531 produced the compounds didethio-11a-methylthioemestrin, 7′-*epi*-didethio-11a-methylthioemestrin, 2″-desmethyl-MPC1001F (**137**), emestrin (**138**), dethiosecoemestrin (**139**) and emestrin H (Table 4). These compounds showed cytotoxic properties against Huh7.5 cells with IC_50_ values ranging from 0.25 to 19 µmol/L (Lv et al. 2023). The hydrothermal vent sediment fungus *Aspergillus terreus* CXX-158-20 produced the cytotoxic compounds asterresin A and D (**140**) and giluterrin (**141**). These had IC_50_ values ranging from 3.96 ± 1.44 to 88.89 ± 9.70 µmol/L against the A549, Namalwa, U266, MCF-7, and MDA-MB-231 cancer cells (Wei et al. 2023). The fungus *Aspergillus aculeatinus* WHUF0198 produced the compounds aculeaquamide A (**142**) and aculeaxanthone C (**143**), which had strong cytotoxic properties against Bel-7402 cell line. IC_50_ values of 3.3 and 1.96 µmol/L were obtained, respectively (Wu et al. 2022, 2023). Figure 4 shows the most active compounds isolated from sediment-derived *Aspergillus*.

**Table 4.** Compounds from *Aspergillus* obtained from deep-sea sediments.

Source	Region/country	Media	Fungi	Compound	Cancer cell line	Activity (IC_50_)	Reference
Deep-sea sediment	Okinawa Trough	Potato juice, glucose	*Aspergillus puniceus* SCSIO z021	Austocystin A, K, L, F B, D, H (**124–130**), M, N, (1′*R*,2′*S*)-F02ZA-1593B2 (**131**), (1′*R*,2′*R*)-compound V (**132**), 8-*O*-methyldemethylsterigmatocystin (**133**), 8-*O*-methyldihydrodemethylsterigmatocystin, versicolorin B (**134**)	Vero	0.60–60 µmol/L	Liang et al. (2021)
Deep-sea sediment	South China Sea	Rice, cornsyrup, peptone, yeast powder, MSG	*Aspergillus nidulans* SD-531	Didethio-11a-methylthioemestrin, 7′-*epi*-didethio-11a-methylthioemestrin, and 2″-desmethyl-MPC1001F (**137**), emestrin (**138**), dethiosecoemestrin (**139**), emestrin H	Huh7.5	0.25–19 µmol/L Sorafenib: 8.2 µmol/L	Lv et al. (2023)
Hydrothermal vent sediment	Taiwan of China	Rice medium	*Aspergillus terreus* CXX-158-20	Asterresin A, D (**140**), giluterrin (**141**)	A549, Namalwa, U266, MCF-7, MDA-MB-231	3.96 ± 1.44 to 88.89 ± 9.70 µmol/L Doxorubicin: 2.57 ± 0.69 µmol/L 5-Fu: 11.28 ± 0.47 µmol/L AS_2_O_3_: 2.59 ± 0.01 µmol/L Adriamycin: 34.91 ± 2.50 µmol/L Adriamycin: 1.08 ± 0.11 µmol/L	Wei et al. (2023)
Deep-sea sediment	South China sea	Number II fungus liquid medium	*Aspergillus aculeatinus* WHUF0198	Aculeaquamide A (**142**), aculeaxanthone C (**143**)	Bel-7402	3.3 µmol/L, 1.96 µmol/L	Wu et al. (2022, 2023)

### 3.4. Others

**Figure 5.**
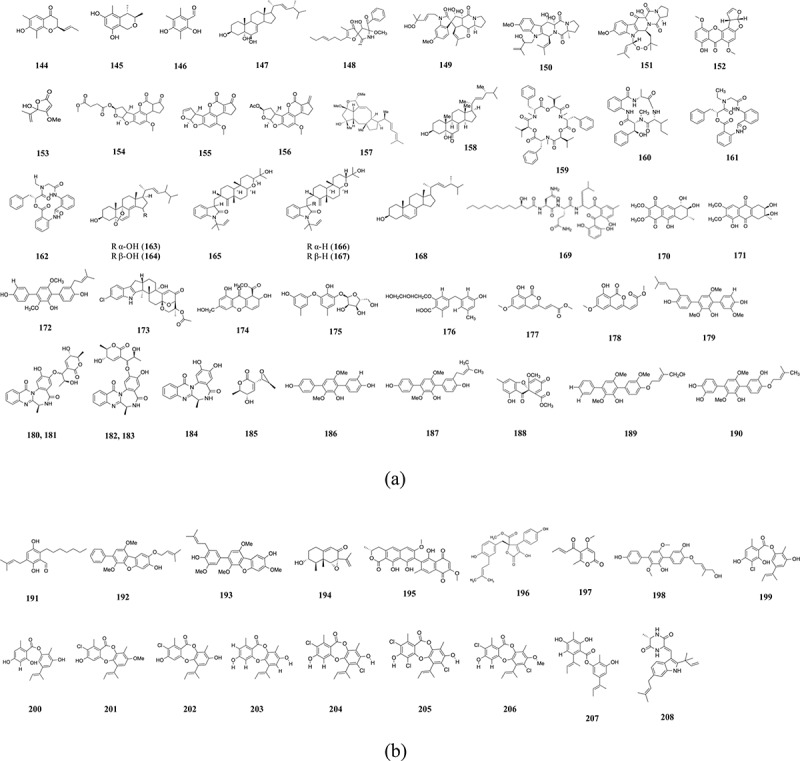
Compounds (**144–208**) isolated from other *Aspergillus* species. (a) Compounds (**144–190**). (b) Compounds (**191–208**).

Five compounds were isolated from the fungus *Aspergillus fumigatus* KMM 4631 associated with a soft coral, namely verruculogen, cyclotryprostatin A, B, 12,13-dihydroxyfumitremorgin C, and fumitremorgin C. These had cytotoxic activity towards Erlich carcinoma tumour cells, with IC_50_ values ranging from 20 to 50 µg/mL (Afiyatullov et al. 2004). Notoamides A–C were isolated from the fungus *Aspergillus* sp. which was obtained from a mussel. They showed moderate cytotoxicity with IC_50_ values of 22–52 µg/mL against HeLa and L1210 cells (Kato et al. 2007). The deep-sea fungus *Aspergillus sydowi* YH11-2 produced eight compounds which showed varying degrees of cytotoxicity against the P388 cell line. It was the compounds (2*R*)-2,3-dihydro-7-hydroxy-6, 8-dimethyl-2-[(*E*)-prop-1-enyl] chromen-4-one (**144**) and 2, 4-dihydroxy-3, 5, 6-trimethylbenzaldehyde (**146**) which showed strong cytotoxicity with IC_50_ of 0.14 and 0.59 µmol/L (Figure 5a) (Table 5). The compounds (3*R*, 4*S*)-3,4,5-trimethylisochroman-6,8-diol (**145**) and cerevisterol (**147**) also had cytotoxic properties (Tian et al. 2007). The fungus *Aspergillus fumigatus* OUPS-T106B-5, obtained from the marine fish *Mugil cephalus*, produced the cytotoxic compound cephalimysin A (**148**). This compound had cytotoxic properties against the cancer cells P388 and HL-60, with IC_50_ values of 15.0 and 9.5 nmol/L, respectively (Yamada et al. 2007).

**Table 5.** Compounds from other *Aspergillus.*

Source	Region/country	Media	Fungi	Compound	Cancer cell line	Activity (IC_50_)	Reference
Deep sea	China	PDB	*Aspergillus sydowii* YH11-2	2,3,5-trimethyl-6-(3-oxobutan-2-yl)-4 H-pyran-4-one, (2*R*)-2,3-dihydro-7-hydroxy-6, 8-dimethyl-2-[(*E*)-prop-1-enyl] chromen-4-one (**144**), (3*R*, 4*S*)-3, 4, 5-trimethylisochroman-6,8-diol (**145**), 5-[(2*S*, 3*R*)-3-hydroxybutan-2-yl]-4- methylbenzene-1,3-diol, (3*R*, 4*S*)-6, 8- dihydroxy-3, 4, 5-trimethylisochroman-l-one, 2, 4-dihydroxy-3, 5, 6-trimethylbenzaldehyde (**146**), (17*R*)-17-methylincistererol, cerevisterol (**147**)	P388	0.12–96.4 µmol/L CDDP: 0.039 µmol/L	Li et al. (2017)
Marine fish *Mugil cephalus*	-	Soluble starch, casein	*Aspergillus fumigatus* OUPS-T106B-5	Cephalimysin A (**148**)	P388, HL-60	15.0, 9.5 nmol/L	Yamada et al. (2007)
Holothurian *S. japonicus*	Lingshan Island, Qingdao, China	Maltose, mannitol, glucose, MSG, yeast extract paste, maize paste	*Aspergillus fumigatus*	Spirotryprostatins C-E (**149**), two derivatives of fumitremorgin B (**150**), 13-oxoverruculogen (**151**)	MOLT-4, A549, HL-60, BEL-7420	1.9–125.3 µmol/L VP16: 0.003–1.400 µmol/L	Wang et al. (2008)
Underwater sample	Pacific Ocean	Maltose, mannitol, glucose, MSG, yeast extract paste, corn steep liquor	*Aspergillus versicolor* CXCTD-06-6a	5- methoxysterigmatocystin (**152**)	A-549, HL-60	3.86, 5.32 µmol/L	Cai et al. (2011b)
Ascidian *Eudistoma vannamei*	Taíba Beach, Ceará state, Brazil	PDB	*Aspergillus* sp.	(*R*)-mellein, *cis*-4-hydroxymellein (**153**), *trans*-4-hydroxymellein, penicillic acid	MDA-MB-435, HCT-8	4.43 to >25.0 µg/mL Doxorubicin: 0.48, 0.04 µg/mL	Montenegro et al. (2012)
Root of mangrove plant *H. tiliaceus*	Wenchang, Hainan Province of China	Glucose, maltose, mannitol, MSG, yeast extract, corn steep liquor	*Aspergillus flavus* 092008	Aflatoxin B_2b_, aflatoxin B_1_, 8-acetoxyaflatoxins B_2_ (**154–156**)	A549, K562, L-02	2.0–6.4 µmol/L	Wang et al. (2012)
Zoanthid *Zoanthus* sp.	Ayamaru Cape, Amami Island, Kagoshima Prefecture Japan	Starch, peptone	*Aspergillus* sp.	Ophiobolin O, 6-*epi*-ophiobolin O (**157**), K, ophiobolin G, H, K	P388	4.7–105.7 µmol/L Vincristine: 120 µmol/L	Zhang et al. (2012)
Branch *Bruguiera gymnoihiza* (Linn.) Savigny	South China Sea in Guangxi Province	PDB	*Aspergillus terreus* (No. GX7-3B)	3β,5α-dihydroxy-(22*E*,24*R*)-ergosta-7,22-dien-6-one (**158**), 3β,5α,14α-trihydroxy-(22*E*,24*R*)-ergosta-7, 22-dien-6-one, beauvericin (**159**)	MCF-7, A459, Hela, KB	0.68–27.1 µmol/L Epirubicin: 0.05–1.07 µmol/L	Deng et al. (2013)
Gorgonian *Echinogorgia aurantiaca*	Sanya, Hainan Province, China	D-sorbitol, yeast extract, L-lysine, maltose	*Aspergillus tereus* SCSGAF0162	Asperterrestide A (**160**)	U937, MOLT-4 Taxol: 1.9, 1.8 nmol/L	6.4, 6.2 µmol/L	He et al. (2013)
*Xenograpsus testudinatus*	Taiwan Kueishantao of China	Sucrose, yeast extract, malt extract	*Aspergillus clavatus* C2WU	Clavatustides A, B (**161, 162**)	HepG2, SMMC-7721, Bel-7402, L02	15 µg/mL	Jiang et al. (2013)
Mangrove plant *Avicennia marina*	Hainan, China	Dextrose, malt extract, peptone	*Aspergillus niger* MA-132	Nigerasterols A, B (**163, 164**)	HL60, A549	0.30 ± 0.01–5.41 ± 0.02 µmol/L Adriamycin: 0.11 ± 0.01, 0.43 ± 0.01 µmol/L	Liu et al. (2013)
Sea urchin *Anthocidariscrassispana*	Tanabe Bay, Wakayama, Japan	D-glucose, peptone, yeast extract	*Aspergillus versicolor*	Anthcolorins B, C, D (**165–167**), A, E, F	P388	2.2–26.7 µmol/L 5-fluorouracil: 1.2 µmol/L	Nakanishi et al. (2013)
Marine solar saltern	Weihai, China	PDB	*Aspergillus* sp. nov. F1	Ergosterol (**168**), rosellichalasin, cytochalasin E	A549, Hela, BEL-7402, RKO	3.3 ± 0.5– 8.5 ± 3.4 µmol/L	Xiao et al. (2013)
Marine submerged decaying wood	–	Semi-solid culture broth	*Aspergillus* sp.	Asperphenins A (**169**), B	K562, RKO; SNU-638, SK-HEP-1, MDA-MB-231	0.8–9.7 µmol/L Etoposide: 0.3–10.1 µmol/L	Liao et al. (2017); Elsbaey et al. (2020)
Coral *Galaxea fascicularis*	Port Dickson, Malaysia	Czapek’s medium	*Aspergillus tritici* SP2-8-1	4-methyl-candidusin A, aspetritone A, B (**170, 171**), 3,4-dimethyl-3”-prenylcandidusin A, 4-methyl-3”-prenylcandidusin A, candidusin A, 3-prenylterphenyllin (**172**), terphenyllin, 3-hydroxyterphenyllin, 3-hydroxy-4”-deoxyterphenyllin, 3”-prenylterphenyllin, emodin, 3-hydroxy-2-hydroxymethyl-1-methoxyanthracene-9,10-dione, 1,2,3-trimethoxy-7-hydroxymethylanthracene-9,10-dione	HeLa, A549, HepG2	2.10 ± 0.20–45.63 ± 1.79 µmol/L Doxorubicin: 0.09 ± 0.01–1.06 ± 0.07 µmol/L	Wang et al. (2017)
Unidentified colonial ascidian	Shikotan Island, Pacific Ocean	Rice, yeast extract	*Aspergillus* sp. KMM 4676	Asperindole A (**173**)	PC-3, LNCaP, 22Rv1	69.4, 47.8, 4.86 µmol/L Docetaxel: 15.4, 3.8, 12.7 nmol/L	Ivanets et al. (2018)
Seawater	West Pacific Ocean	–	*Aspergillus sydowii* strain C1-S01-A7	2-hydroxy-6-formyl-vertixanthone, 12-*O*-acetyl-sydowinin A, AGI-B4 (**174**), questin, yicathin C, emodin	A549, HepG2, HeLa	8.1 ± 1.3–42.3 ± 0.6 µmol/L Doxorubicin: 0.1 ± 0.0–0.6 ± 0.1 µmol/L	Wang et al. (2018a)
Marine water	Sea of Xiamen, China	Rice medium	*Aspergillus sydowii* FNA026	cordyol C-3-*O-α*-d-ribofuranoside (**175**), 7-ethyldiorcinol, 3-hydroxydiorcinol, diorcinol, glyceryl diorcinolic acid (**176**), cordyol C, aspergilol E	A549, U937, HL-60, K562	3.36 ± 0.68– 23.03 ± 1.34 µmol/L Dox: <0.125– 0.49 ± 0.08 µmol/L	Wang et al. (2018b)
Mangrove *Acanthus ilicifolius* leaves	Dongzhaigang Mangrove National Nature Reserve, Hainan Island, China	Rice medium	Aspergillus sp. HN15-5D	Aspergisocoumrin A, B (**177, 178**)	MDA-MB-435, HepG2, HCT116, H460, MCF10A	4.98 ± 0.74 to >50 µmol/L Epirubicin: 0.12 ± 0.01–0.37 ± 0.05 µmol/L	Wu et al. (2018)
Leaves of mangrove plant *Avicennia marina*	Red sea coast, Hurghada, Egypt	Rice medium	Aspergillus sp. AV-2	Flavoglaucin (**179**)	Caco-2	2.87 µmol/L	Elissawy et al. (2019)
Coral *Dichotella gemmacea*	Lingao, Hainan Province, China	Soluble starch, peptone	*Aspergillus ochraceus* LCJ11-102	Ochrazepines A–D (**180–183**), 2-hydroxycircumdatin C (**184**), aspyrone (**185**)	MV-4-11, K562, A673, U87, A549, N87, H1299, HUCCT1, B16F10, Karpass299, U251, Hep3B, A431, 143B, MKN-45, H1975, HL60, DU145, SPC-A1, HEK-293F, L02	2.54 to >100 µmol/LAdramycin: 0.02–17.58 µmol/L	Fan et al. (2019)
Deep sea	Atlantic Ocean	Rice medium	*Aspergillus candidus*	Terphenyllin (1**86**), prenylterphenyllin (**187**)	Hela, Eca-109, Bel-7402, PANC-1	5.5–9.4 µmol/L	Lin et al. (2019)
Soft coral *Sinularia* sp.	South China Sea	Rice medium	*Aspergillus* sp.	(-)-bis-dechlorogeodin (**188**)	Jurkat, A549, HeLa	10.69, 10.69, 3.56 µmol/L Adriamycin: 0.53, 0.60, 0.86 µmol/L	Said et al. (2019)
Root of *Rhizophora apiculata* Blume	Sanya Bailu Park of Hainan Province, China	Mannitol, MSG, maltose, yeast extract, glucose, corn steep liquor	*Aspergillus candidus* LDJ-5	Prenylterphenyllins F–J (**189–191**), prenylcandidusins D, F, E, G (**192, 193**)	L-02, MGC-803, HCT-116, BEL-7402, A549, SH-SY5Y, HeLa, U87, K562, HL-60	0.4 to >50.0 µmol/L Adriamycin: 0.02–0.6 µmol/L	Zhou et al. (2020)
Fresh inner tissue *Meretrix meretrix*	Hailing Island, Yang-jiang, China	Rice medium	*Aspergillus flavus* BB1	Sporogen-AO (**194**), phomaligol G, H	A549, H1299, SK-BR-3, HCT-116	0.13–65.53 µmol/L	Liu et al. (2021)
Leaves of mangrove plant *Avicennia marina*	–	–	*Aspergillus sydowii* #2B	(±)-pyrenocine S, xanthoradone A (**195**), (+)-3,3’,7,7’,8,8’ -hexahydroxy-5,5’ -dimethyl-bianthra-quinone, butyrolactone-I (**196**), pyrenocine A (**197**), (±)-pyrenocine E	VCaP	1.92 ± 0.82– 33.36 ± 1.42 µmol/L	Wang et al. (2021)
Root of *Rhizophora apiculata* Blume	Sanya Bailu Park of Hainan Province, China	Mannitol, MSG, maltose, yeast extract, glucose, corn steep liquor	*Aspergillus candidus* LDJ-5	Asperterphenyllin A-G (**198**)	L-02, MGC-803, HCT-116, BEL-7402, A549, SH-SY5Y, Hela, U87, HO8910	0.4 to >50 µmol/L	Zhou et al. (2021)
Seawater; Deep-sea shrimp *Rimicaris* sp.	Socheongcho Ocean Research Station, Korea; Indian Ocean	Bennett’s broth medium	*Aspergillus unguis* 158SC-067; *Aspergillus unguis* IV17-109	Unguidepside C (**199**), decarboxyunguidepside A (**200**), aspersidone B (**201**), 2-chlorounguinol (**202**), unguinol (**203**), 3,10-dichlorounguinol (**204**), nidulin, nornidulin (**205**), aspersidone (**206**), agonodepside B (**207**), agonodepside C, agonodepside A, guisinol, folipastatin, emeguisin A	ACHN, NCI-H23, PC-3, NUGC-3, MDA-MB-231, HCT-15	2.5–46.9 µmol/L Adriamycin: 0.12–0.16 µmol/L	Anh et al. (2022)
Deep sea	China	Rice, millet	*Aspergillus chevalieri* MCCC M23426	Neoechinulin B, D (**208**) 5-prenylcryptoechinulin A, 9-*epi*-didehydroechinulin, (12*S*,28*S*,31*R*)-cryptoechinulin D, neoechinuline, cryptoechinulin A, 7-prenylneoechinulin B, variecolorin H, cryptoechinulin C, neoechinulin A	MKN1	4.6 to >100 µmol/L Cisplatin: 8.8 µmol/L	Lv et al. (2022)

Terpeptin A and B were isolated from the mangrove endophytic fungus *Aspergillus* sp. (W-6). These compounds showed cytotoxicity against A-549 cell line with IC_50_ values of 23.3 and 28.0 µmol/L, respectively (Lin et al. 2008). The fungus *Aspergillus* sp. was isolated from the mussel *Mytilus edulis galloprovincialis*. The compound notoamide I was obtained from this fungus and showed cytotoxicity against HeLa cells with IC_50_ value of 21 µg/mL (Tsukamoto et al. 2008). New compounds were isolated from the fungus *Aspergillus fumigatus* associated with a holothurian. These were identified as three spirotryprostatuns C, D and E (**149**), a derivative of fumitremorgin B (**150**) and 13-oxoverruculogen (**151**). The tetrazolium (MTT) and sulforhodamine B (SRB) assays revealed that they were cytotoxic against MOLT-4, A549, HL-60, and BEL-7420 cell lines, with IC_50_ values ranging from 1.9 to 125.3 µmol/L (Wang et al. 2008). The fungus *Aspergillus versicolor* MST-MF495 was isolated from the beach sand of Cottesloe, Western Australia. Two new compounds were obtained from this isolate, namely, cotteslosins A and B which had cytotoxic properties against the human melanoma MM418c5, prostate DU145, and breast T47D cell lines (IC_50_ values 66–94 µg/mL) (Fremlin et al. 2009). Aspernolide A, isolated from the fungus *Aspergillus terreus*, obtained from a soft coral, showed weak cytotoxicity against the five cancer cell lines H460, ACHN, Calu, Panc1, and HCT-116 with the IC_50_ values of >88, >103, >147, >130, and >121 µmol/L, respectively (Parvatkar et al. 2009). Wang et al. (2010) isolated *Aspergillus fumigatus* WFZ-25 from the holothurian *S. japonicus*. Two metabolites, pseurotin A and A2, were cytotoxic against A549 and HL-60, with IC_50_ values ranging from 48.0 to 67.0 µmol/L.

The compound 5-methoxysterigmatocystin (**152**) was isolated from a deep-sea fungus *Aspergillus versicolor* CXCTD-06-6a. It showed cytotoxicity against A-549 and HL-60 with IC_50_ values of 3.86 and 5.32 µmol/L, respectively (Cai et al. 2011b). The mangrove endophyte, *Aspergillus tubingensis* GX1-5E, produced the compounds TMC 256 A1, rubasperone D, rubrofusarin B, flavasperone which had cytotoxic properties against a panel of cancer cells, namely MCF-7, MDA-MB-435, Hep3B, Huh7, SNB19, and U87 MG. The IC_50_ values obtained ranged from 19.92 to >100 µmol/L (Huang et al. 2011). *Aspergillus niger* MA-132 was isolated from the mangrove plant *Avicennia marina*. The compounds nigerapyrone B, D, E, and asnipyrones A had cytotoxic properties against the cancer cells DU145, HeLa, HepG2, MCF-7, NCI-H460, MDA-MB-231, SW1990, and A549. The IC_50_ values ranged from 38 to 121 µmol/L (Liu et al. 2011). Oxalicine B, derived from the sea urchin fungus *Aspergillus fumigatus* OUPS-N138, showed moderate cytotoxicity (IC_50_ = 55.9 µmol/L) against P388 cells (Kitano et al. 2012). The fungus *Aspergillus* sp. associated with an ascidian produced the four compounds (*R*)-mellein, *cis*-4-hydroxymellein (**153**), *trans*-4-hydroxymellein, and penicillic acid. These compounds were cytotoxic against the cancer cell lines MDA-MB-435, and HCT-8, with IC_50_ values ranging from 4.43 to >25.0 µg/mL (Montenegro et al. 2012). Aflatoxins (**154–156**) were responsible for the cytotoxic activity of the mangrove endophyte *Aspergillus flavus* 092008 against K562, L-02, and A549. The IC_50_ values obtained ranged from 2.0 to 6.4 µmol/L (Wang et al. 2012). The sesterterpenes ophiobolin O, 6-*epi*-ophiobolin O (**157**), K, ophiobolin G, H, and K were obtained from the fungus *Aspergillus* sp. associated with a zoanthid. These showed cytotoxic activities against the mouse leukaemia cell-line P388, with IC_50_ values 4.7–105.7 µmol/L (Zhang et al. 2012). The fungus *Aspergillus fumigatus*, isolated from the zoanthid *Zoanthus* sp., produced the compounds 2-(3,3-dimethylprop-1-ene)-costaclavine, 2-(3,3-dimethylprop-1-ene)-epicostaclavine, costaclavine and fumgaclavine C. These compounds displayed weak cytotoxicity against the mouse leukaemia P388 cells, with IC_50_ values 64.9–218.8 µmol/L (Zhang et al. 2012).

The mangrove endophyte *Aspergillus terreus* (No. GX7-3B) produced six cytotoxic compounds, namely, anhydrojavanicin, 8-*O*-methylbostrycoidin, 3β,5α-dihydroxy-(22*E*,24*R*)-ergosta-7,22-dien-6-one (**158**), 3β,5α,14α-trihydroxy-(22*E*,24*R*)-ergosta-7,22-dien-6-one, NGA0187, and beauvericin (**159**). These decreased the cell viability of MCF7, A549, Hela, and KB cells, at IC_50_ values of 0.68–27.1 µmol/L (Deng et al. 2013). The fungus *Aspergillus terreus* SCSGAF0162, associated with a gorgonian, produced the cytotoxic compound asperterrestide A (**160**) against the cancer cells U937 and MOLT-4. IC_50_ values of 6.4 and 6.2 µmol/L were obtained, respectively (He et al. 2013). Two cyclodepsipeptides, clavatustides A and B (**161, 162**), were obtained from the hydrothermal vent crab-associated fungus *Aspergillus clavatus* C2WU. These prevented the proliferation of HepG2 cells by increasing the number of cells in the G1 phase and decreasing the S phase (Jiang et al. 2013). The fungus *Aspergillus niger* MA-132, isolated from the mangrove plant produced two cytotoxic sterols, nigerasterols A and B (**163, 164**). These had anti-proliferative effects on the cells HL60 and A549, with IC_50_ values ranging from 0.30 ± 0.01 to 5.41 ± 0.02 µmol/L (Liu et al. 2013). *Aspergillus versicolor* was isolated from the sea urchin *Anthocidaris crassispina*. It produced the compounds anthcolorins B, C, D (**165–167**), A, E, and F which had growth inhibition properties against the P388 cells, with IC_50_ values ranging from 2.2 to 26.7 µmol/L (Nakanishi et al. 2013). *Aspergillus* sp. nov. F1 was isolated from the marine solar saltern in China. This fungus produced compounds that had cytotoxic properties against A549, Hela, BEL-7402, and RKO cells (IC_50_: 3.3 ± 0.5 to 78.5 ± 3.4 µmol/L). The compounds responsible for this property were ergosterol (**168**), rosellichalasin, and cytochalasin E (Xiao et al. 2013).

*Aspergillus versicolor* ZBY-3 was isolated from a deep-sea water sample in the southeast Pacific by Dong et al. (2014). The compounds, cyclo(D-Pro-D-Phe), cyclo(D-Tyr-D-Pro), phenethyl 5-oxo-L-prolinate, cyclo(L-Ile-L-Pro), cyclo(L-Leu-L-Pro) and 3β,5α,9α-trihydroxy-(22*E*,24*R*)-ergosta-7,22-dien-6-one, displayed cytotoxic properties against the cancer cell lines K562, HL-60, HeLa, and BGC-823, with IC_50_ ranging from 39.5 to >150 µmol/L. The marine fungi *Aspergillus* sp., isolated from the gut of the marine isopod *Ligia oceanica*, produced the cytotoxic compound aspochalasin V. This compound had IC_50_ values of 30.4 and 39.2 µmol/L against PC3 and HCT116 cancer cell lines (Liu et al. 2014). *Aspergillus flavus* OUCMDZ-2205, isolated from the prawn *Penaeus vannamei*, produced four cytotoxic compounds, (2*R*,4b*R*,6a*S*,12b*S*,12c*S*,14a*S*)-4b-deoxyβ-aflatrem, (2*R*,4b*S*,6a*S*,12b*S*,12c*R*)-9-isopentenyl paxilline, β-aflatrem, paspalinine against MCF-7 and A549 cells. The IC_50_ values obtained ranged from 18 to 30 µmol/L (Sun et al. 2014). The compound clavatuside B was isolated by Ye et al. (2014) from *Aspergillus clavatus* C2WU. This isolate was obtained from *Xenograpsus testudinatus* which was found in the sulphur-rich hydrothermal vents of Taiwan Kueishantao of China. The compound clavatustide B inhibited the growth of eight cancer cell lines HepG2, SMMC-7721, Bel-7402, Panc-1, SW-480, WERI-Rb-1, and PC3 (IC_50_: 15–20 µg/mL).

A new compound, 4-(3-hydroxyphenyl)-3-methoxyquinolin-2(1H)-one, was isolated from the seawater fungus *Aspergillus versicolor* Y31-2. It had moderate cytotoxicity against MCF-7 and SMMC-7721 with IC_50_ of 16.6 and 18.2 µmol/L (Li et al. 2016b). Another *Aspergillus* sp. 2C1-EGY was isolated from the soft coral *Sinularia* sp. by Abd El-Hady et al. (2017). The fractions showed cytotoxic properties against HCT-116 cancer cells. Aspersymmetide A was isolated from the fungus *Aspergillus versicolor* TA01-14. This fungus was isolated from the gorgonian *Carijoa* sp. The compound was cytotoxic against NCI-H292 and A431 cells at a concentration of 10 µmol/L (Hou et al. 2017). Asperphenin A (**169**), was obtained from *Aspergillus* sp. isolated from a marine submerged decaying wood. It showed strong cytotoxicity against RKO cells with IC_50_ of 0.84 ± 0.26 µmol/L (Liao et al. 2017; Bae et al. 2020). *Aspergillus tritici* SP2-8-1 was obtained from the coral *Galaxea fascicularis*. It produced the three compounds aspetritone A, B (**170, 171**) and 3-prenylterphenyllin (**172**) which showed stronger cytotoxic activities with IC_50_ <5 µmol/L against the cancer cells HeLa, A549, and HepG2 (Wang et al. 2017).

The fungus *Aspergillus versicolor* LZD-14-1 was isolated from the gorgonian *Pseudopterogorgia* sp. (LZD-14). It produced two cytotoxic compounds. Versiquinazoline P, Q displayed weak cytotoxicity against A549 (IC_50_ >10 µmol/L) but had inhibitory activities against thioredoxin reductase (TrxR) with IC_50_ values 13.6 ± 0.6 and 12.2 ± 0.7 µmol/L (Cheng et al. 2018). Handayani et al. (2018b) isolated *Aspergillus sydowii* from the mangrove plant *Sonneratia alba*. Its fungal extract had low percentage viability with T47D. The anticancer property of L-asparaginase, produced by the marine fungi *Aspergillus terreus* obtained from the Red Sea, was investigated by Hassan et al. (2018). It was tested against the cell lines HCT-116, HepG2, and MCF-7 and an IC_50_ of 3.79–12.6 µg/mL was obtained. Asperindole A (**173**) was obtained from the fungus *Aspergillus* sp. KMM 4676, which was isolated from an unidentified colonial ascidia-derived. Asperindole A had cytotoxicity against the three cell lines PC3, LNCaP, and 22Rv1 with IC_50_ of 69.4 µmol/L, 47.8 µmol/L, and 4.86 µmol/L (Ivanets et al. 2018). The compound aspochalasins D was isolated from *Aspergillus* sp., which was found in the gut of the marine isopod *Ligia oceanica*. It showed strong cytotoxicity (IC_50_ 11.14 µmol/L) against the prostate cancer PC3 cell line. Four other compounds were also isolated but these showed weak activity (Li et al. 2018b). The isolate *Aspergillus sydowii* strain C1-S01-A7 was isolated from the deep seawater of the West Pacific Ocean. Six cytotoxic compounds were reported from this fungus, namely 2-hydroxy-6-formyl-vertixanthone, 12-*O*-acetyl-sydowinin A, AGI-B4 (**174**), questin, yicathin C, and emodin. The IC_50_ values obtained ranged from 8.1 ± 1.3 to 42.3 ± 0.6 µmol/L, against A549, HepG2, and HeLa (Wang et al. 2018a). Seven compounds, cordyol C-3-*O-α*-d-ribofuranoside (**175**), 7-ethyldiorcinol, 3-hydroxydiorcinol, diorcinol, glyceryl diorcinolic acid (**176**), cordyol C, and aspergilol E, were obtained from the marine water fungus *Aspergillus sydowii* FNA026. These displayed selective cytotoxicity against the cancer cell lines A549, U937, HL-60, and K562, with IC_50_ values ranging from 3.36 ± 0.68 to 23.03 ± 1.34 µmol/L (Wang et al. 2018b). The two compounds aspergisocoumrins A, B (**177, 178**), isolated from the mangrove endophyte *Aspergillus* sp. HN15-5D, showed cytotoxic properties against the cancer cell lines MDA-MB-435, HepG2, HCT116, H460, and MCF10A. The IC_50_ values obtained ranged from 4.98 ± 0.74 to >50 µmol/L (Wu et al. 2018). Bispyrrolidinoindoline diketopiperazines were obtained from the broth of the marine shrimp fungus *Aspergillus* sp. DX4H and had weak cytotoxic properties against PC3 cell line, at a concentration of 20 µg/mL (Xu et al. 2018).

Flavoglaucin (**179**) was the most cytotoxic against Caco-2 cells, with IC_50_ value of 2.87 µmol/L. This compound was produced by the mangrove endophyte *Aspergillus* sp. AV-2 (Elissawy et al. 2019). Six compounds were isolated from the coral-associated fungus *Aspergillus ochraceus* LCJ11-102. The compounds ochrazepine A (**180**) and aspyrone (**185**) showed broad spectrum cytotoxicity against the 26 human cancer cells involved in the study. Compounds ochrazepines B, D (**181, 183**) and 2-hydroxycircumdarin C (**184**) showed selective cytotoxicity against U251 while ochrazepine C (**182**) was active against A673, U87, and Hep3B. The IC_50_ values obtained ranged from 2.54 to >100 µmol/L (Fan et al. 2019). The fungi *Aspergillus awamori* and *Aspergillus niger* were isolated from the coral *Parazoanthus axinella* and the tunicate *Microcosmus vulgaris*, respectively. Their extracts were tested against HCT-116 and showed strong cytotoxic activities with IC_50_ values of 3.13 ± 0.58 µg/mL and 4.428 ± 0.60 µg/mL (Heydari 2019). The marine-derived fungus, *Aspergillus versicolor*, was isolated from a clam and produced seven cytotoxic compounds that were evaluated against a panel of cancer cell lines. The compounds were named 6,6′-oxybis(1,3,8-trihydroxy-2-((*S*)-1-methoxyhexyl) anthracene-9,10-dione, 6,6′-oxybis(1,3,8-trihydroxy-2-((*S*)-1-hydroxyhexyl) anthracene-9,10-dione, 1’-*O*-methylaverantin, averantin, averythrin, sterigmatocystin, and variecoxanthone A. These compounds were tested against a panel of cancer cell lines, namely, A-549, SK-OV-3, SK-MEL-2, XF-498, and HCT-15. The IC_50_ values ranged from 11.25 to >30 µg/mL (Li et al. 2019a). Cytotoxic terphenyllin (**186**) and prenylterphenyllin (**187**) were isolated from deep-sea derived *Aspergillus candidus*. These had anti-proliferative properties against Hela, Eca-109, Bel-7402, and PANC-1 with IC_50_ values of 5.5–9.4 µmol/L (Lin et al. 2019). Another *Aspergillus* sp., isolated from the soft coral *Sinularia* sp., produced the compound (-)-bis-dechlorogeodin (**188**). The latter was cytotoxic against the cancer cells Jurkat, A549, and HeLa cells with IC_50_ values of 10.69, 10.69, and 3.56 µmol/L, respectively (Said et al. 2019). The fruit of the mangrove plant *Avicennia marina* had as endophyte *Aspergillus versicolor*. This fungus produced allantopyrone E which was cytotoxic against HeLa cells with IC_50_ 50.97 µmol/L (Elsbaey et al. 2020).

The compounds aspermytin A, versicomide A, versicoloid A, isochaetominines A–C, and 14-*epi*-isochaetominine C were isolated from a decaying wood fungus *Aspergillus* sp. These compounds showed IC_50_ values of 13 to >100 µmol/L against the cancer cells A549, K562 (Park et al. 2020). Ukwatta et al. (2020) isolated *Aspergillus terreus* from the mangrove plant *Bruguiera gymnorrhiza*. The pure compound, cowabenzophenone A, was cytotoxic against HCT-116 cells with IC_50_ value of 10.1 µmol/L. The cytotoxic effect of aspergillipeptide D on Vero cells was examined by Wang et al. (2020c). An IC_50_ value of 208.723 ± 9.717 µmol/L was obtained. This compound was obtained from the fungus *Aspergillus* sp. SCSIO 41501 is associated with a gorgonian. A reduction in cell viability was observed at concentration >25 µmol/L. Out of the nine compounds isolated from the mangrove root endophyte *Aspergillus candidus* LDJ-5, prenylterphenyllin F, H, J (**189–191**) displayed the best activity. Prenylterphenyllin H (**190**) had IC_50_ values of 3.5, 0.7, 0.5, 0.4, 0.6, and 2.0 μmol/L against the cancer cells lines L-02, MGC-803, HCT-116, A549, SH-SY5Y, and HeLa, respectively. The compounds prenylcandidusins E, G (**192, 193**) also showed cytotoxicity (Figure 5b) (Zhou et al. 2020). The compounds asperochratide E–H, notoamide M, sclerotiamide, notoamide A, and aspilactonol E had cytotoxic properties against BV-2 cell line with inhibition range of 50.26%–72.81%. These compounds were isolated from the deep-sea fungus *Aspergillus ochraceus* (Zou et al. 2020).

*Aspergillus flavipes* 297, isolated from seawater, produced flavilanes A and B which had cytotoxic activities against A549, HCT-116, MKN-45, and HepG2 cells. The IC_50_ values obtained ranged from 19.8 ± 0.6 to >50 µg/mL (Chen et al. 2021). The extract obtained from the fungus *Aspergillus unguis* SPMD-EGY, which was obtained from a soft-coral, showed cytotoxic properties against the cancer cells HepG2, MCF-7, RPE-1, and HCT-116. It was during static malt extract media culture that the broth extract showed low IC_50_ value of 6.5 ± 0.43 µg/mL against MCF-7 cells (El-Shahid et al. 2021). A new prenylated indole alkaloid from the seawater-derived fungus *Aspergillus austroafricanus* Y32-2 was isolated by Li et al. (2021). It had cytotoxic activity against HepG2 with IC_50_ value of 30 µg/mL. According to Liao et al. (2021), *Aspergillus ochraceopetaliformis* DSW-2, isolated from seawater, produced the five cytotoxic compounds sclerotiotide M, sclerotiotide B, sclerotiotide F, mactanamide, and cyclo-(L-Pro-L-Tyr). These showed weak cytotoxic properties (IC_50_ > 20 µmol/L) against pancreatic cancer cells HPAC and BXPC3. Liu et al. (2021) isolated three cytotoxic compounds from *Aspergillus flavus* BB1, obtained from the shellfish *Meretrix meretrix*. The compounds, namely, sporogen-AO (**194**), phomaligol G, and H were cytotoxic against the cancer cell lines A549, H1299, SK-BR-3, and HCT-116. The IC_50_ values ranged from 0.13 to 65.53 µmol/L. Scopularide I, isolated from the fungus *Aspergillus sclerotium* SCSIO 41031, which was obtained from soft-coral, displayed cytotoxic properties against human nasopharyngeal carcinoma cell lines HONE1 and HONE1-EBV with IC_50_ values of 13.0 and 10.1 µmol/L, respectively (Long et al. 2021).

The secondary metabolites of three *Aspergillus* species, namely, *A. terreus*, *A. fumigatus*, and *A. flavus*, had cytotoxic properties against the cancer cells Caco-2 and HuH-7. A concentration dependent cytotoxicity was observed with cell rounding, detachment and membrane blipping. A decreased IC_50_ in Caco-2 and HuH-7 cells was observed with treatment with *A. terreus* and *A. fumigatus*. A range of compounds were characterised by GC-MS, including 2,4,6-triphenylpyridin, nizatidin, pyrrolo[1,2]-apyrazine-1,4-dione,hexahydro-3-(2-methylpropyl)], 1-tetradecanamine, *n,n*-dimethyl, 2,4-di-tert-butylphenol, pent-4-enoic acid, 2-(2-hydroxy-3-isobutoxypropyl), hydrazide, 1-dodecanamine, *n,n*-dimethyl, nizatidine, decan-2-yl isobutyl carbonate, 3-t-pentylcyclopentanone, 1-tetradecanamine, *n,n*-dimethyl, 2-methylhexadecan-1-ol, 1-chlorooctadecane, benzenmethanol 3,4,5-trimethoxy (Mohamed et al. 2021). The compounds (±)-pyrenocine S, xanthoradone A (**195**), (+)-3,3’,7,7’,8,8’-hexahydroxy-5,5’-dimethyl-bianthra-quinone, butyrolactone-I (**196**), pyrenocine A (**197**), and (±)-pyrenocine E, were isolated from the mangrove fungus *Aspergillus sydowii* #2B. They showed cytotoxicity against the prostate cancer VCaP cells, with IC_50_ values ranging from 1.92 ± 0.82–33.36 ± 1.42 µmol/L (Wang et al. 2021). Out of the seven compounds isolated from the mangrove endophytic fungus *Aspergillus candidus* LDJ-5, it was asperterphenyllin G (**198**) that had strong cytotoxicity against the nine cancer cell lines used in the study. The IC_50_ value of 0.4 µmol/L was obtained against A549 cells (Zhou et al. 2021).

Two species of *Aspergillus unguis*, namely *A. unguis* 158SC-067 and *A. unguis* IV17-109, were isolated from sea water and the shrimp *Rimicaris* sp., respectively. These fungi produced fourteen compounds with cytotoxic properties against ACHN, NCI-H23, PC-3, NUGC-3, MDA-MB-231, and HCT-15. These compounds were unguidepside C (**199**), decarboxyunguidepside A (**200**), aspersidone B (**201**), 2-chlorounguinol (**202**), unguinol (**203**), 3,1’-dichlorounguinol (**204**), nidulin, nornidulin (**205**), aspersidone (**206**), agonodepside B (**207**), agonodepside A, guisinol, folipastatin, emeguisin A. The IC_50_ values obtained ranged from 2.5 to 46.9 µmol/L (Anh et al. 2022). The gorgonian-derived fungus, *Aspergillus hiratsukae* SCSIO 7S2001, was found to produce the cytotoxic metabolites chevalones I, L, and echinulin. These decreased the cell viability of SF-268, MCF-7, HepG2, and A549 cells. The IC_50_ values obtained were from 12.75 ± 1.43 to 107.31 ± 9.83 µmol/L (Chen et al. 2022). Three tripeptide asterripeptides A–C, obtained from the mangrove endophyte *Aspergillus terreus* LM.5.2., had cytotoxic properties against the cancer cells PC3, MCF-7, DLD-1, and H9C2, with IC_50_ values ranging from 58.3 ± 3.2 to 104.1 ± 3.3 µmol/L (Girich et al. 2022). A mangrove endophyte, *Aspergillus ustus* 094102, produced the compound ustusolate I. This compound had cytotoxic properties against CAL-62 and MG-63 cancer cells, with IC_50_ values of 16.28 ± 1.01 and 10.08 ± 0.04 µmol/L, respectively (Gui et al. 2022). The extract of *Aspergillus flavus*, isolated from the water of El-Qussair, had cytotoxic properties against HepG2, HCT-116, and MCF-7 cells, with IC_50_ values of 62.13, 115.93, and 154.82 µg/mL, respectively. GC-MS analysis of the extract showed that it contained the compounds methylbenzylamine, *n*-heptyl-noctyl, naphthalene, 2,3,6-trimethyl-, octadecanoic acid, ethyl ester, 1,2-benzenedicarboxylic acid, butyl octyl ester, tributyl acetylcitrate, 1,2-benzenedicarboxylic acid, and diisooctyl ester (Khattab et al. 2022). The deep-sea fungus *Aspergillus chevalieri* MCCC M23426 produced the compounds neoechinulin B and D (**208**), which had cytotoxic activity against gastric cancer cells MKN1, with IC_50_ values of 20.7 and 4.6 µmol/L, respectively (Lv et al. 2022). *Aspergillus fumigatus* M580, isolated from the sea cucumber *Colochirus quadrangularis*, produced fumiquinazoline C, D, and J, which had cytotoxic properties against HuH7 and HT-29 cells (Tuan et al. 2022). *Aspergillus flavipes* DS720, isolated from deep seawater, produced indole flavonoid A which had cytotoxic properties against the human tumour cell lines, HeLa, 5637, CAL-62, PATU8988T, A-375, and A-673 (Xu et al. 2022). The four compounds asperopiperazine A, B, (+)-citreosiocoumarine, and (-)-6,8-di-*O*-methylcitreoisocoumarine were obtained from the fungus *Aspergillus* sp. DY001 associated with a tunicate. These compounds had cytotoxic properties against the cancer cells MDA-MB-231 and HCT 116, with IC_50_ values ranging from 15.1 ± 0.1 to 35.0 ± 0.2 µmol/L (Youssef et al. 2022). The compounds demethylincisterol A2 and butyrolactone I were obtained from the fungus *Aspergillus hiratsukae* SCSIO 5Bn_1_003 associated with a soft coral. They had moderate cytotoxic activity against SF-268, HepG-2, MCF-7, and A549 cancer cell lines, with IC_50_ values ranging from 31.03 ± 3.04 to 50.25 ± 0.54 µmol/L (Zeng et al. 2022). The *Aspergillus* sp., obtained from a soft coral and isolated by Elnaggar et al. (2023), produced a new meroterpenoid austalide Z. This compound displayed cytotoxic properties against Caco-2 cell line with moderate IC_50_ value of 51.6 ± 0.88 µg/mL. Figure 5b shows the most active compounds isolated from other *Aspergillus.*

**Figure 6.**
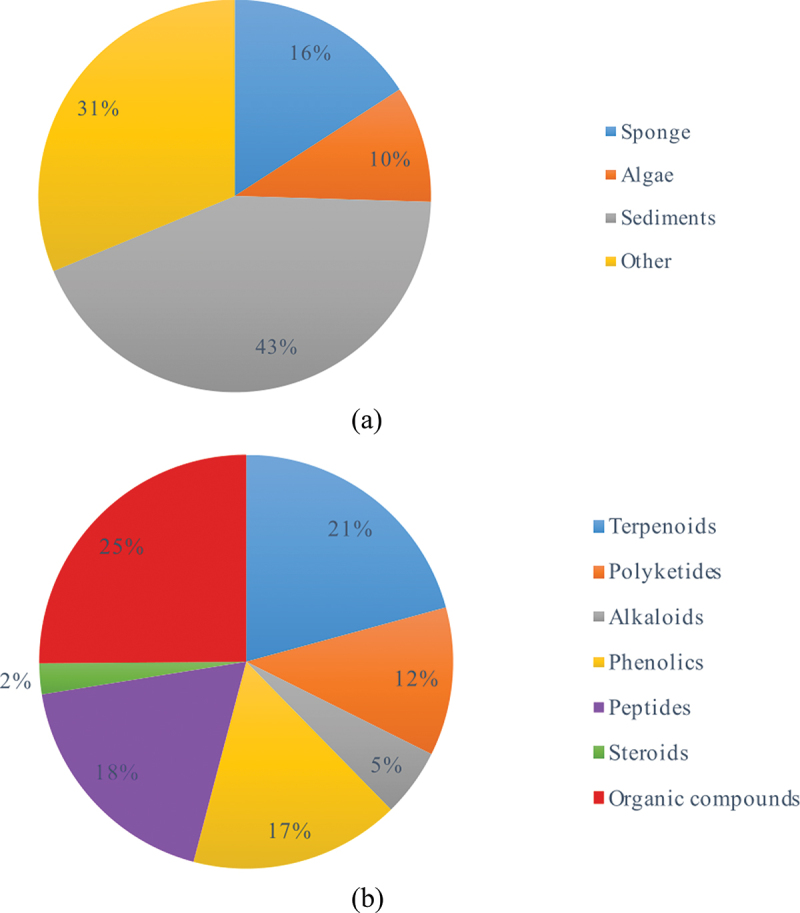
Sources of *Aspergillus*. (a) Sources of *Aspergillus* from which anticancer compounds were reported. (b) Cytotoxic compounds from *Aspergillus*, divided by structural types.

According to Figure 6a, it can be seen that most cytotoxic compounds were derived from *Aspergillus* isolated from sediments (43%), followed by other sources (31%), sponges (16%), and lastly algae (10%). While most *Aspergillus* were isolated from marine sediments, a newly emerging source, the deep-sea sediments, are being considered. Despite the extreme conditions prevailing in this hostile environment, the *Aspergillus* produced structurally unique metabolites with strong cytotoxic properties. Different classes of metabolites were reported in this review (Figure 6b). The 208 compounds belonged to organic compounds (25%), terpenoids (21%), peptides (18%), phenolics (17%), polyketides (12%), alkaloids (5%), and steroids (2%).

## 4. Mechanism of action

Anticancer drugs are classified into two main categories: cytotoxic and targeted agents. Cytotoxic drugs kill the dividing cells by preventing DNA replication and mitosis, whereas targeted agents prevent the uncontrolled proliferation of cancerous cells by interacting with specific molecular proteins involved in cancer growth pathways (Sun et al. 2017). Understanding the mechanism of action is important in drug discovery. The major challenge is designing a drug with high specificity for cancer cells and not normal cells. This specificity will reduce the side effects and lead to better outcomes for the patients (National Cancer Institute 2022). Anticancer drug targets include kinases, tubulin/microtubule, tumour vasculature, cancer stem cells, and monoclonal antibodies (Kumar et al. 2017). Due to the lack of information on the mechanism of action of the molecules highlighted above, this section will be restricted to only available scientific evidence based on published scientific studies.

### 4.1. Aspergillus *associated with marine sponges*

**Figure 7.**
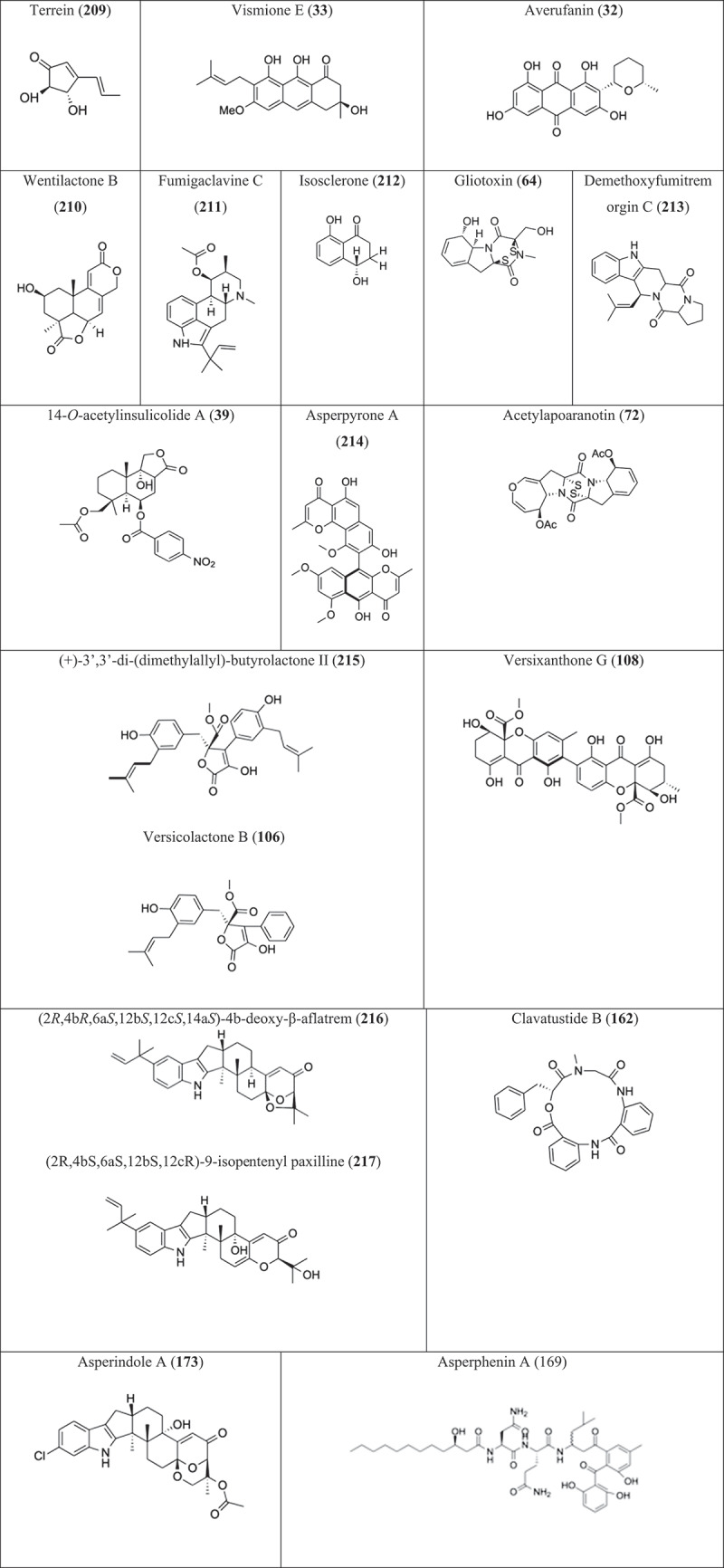
Structure of anticancer compounds.

Terrein (**209**), isolated from the *Aspergillus terreus* PF26, reduced sKOV3 cell migration (Figure 7). It increased early apoptotic cells from 0.99% to 3.02% and late apoptotic cells from 2.19% to 3.71%. Cell cycle arrest at the G2/M phase was observed as well as down-regulation of cyclin B1 and Cdc2. Terrein (**209**) also suppressed the expression of the RNA-binding protein LIN28. The anticancer effects of terrein on the primary hOVCCs, isolated from tumour of three patients, were also evaluated. Reduction in cell viability after treatment with terrein was observed, more effectively than the drug cisplatin. The upregulation of the markers ALDH1, ALDH2, ABCG2, CXCR4, MyD88, and LIN28 in the human ovarian CSLCs was observed (Chen et al. 2014b) (Table 6). Vismione E (**33**) was obtained from the fungus *Aspergillus* sp. 1901NT-1.2.2. It was cytotoxic to MCF-7 cells and caused cell cycle arrest in the G1 phase and a decrease in the number of cells in the first division. Staining with 5-Ethynyl-2’-deoxyuridine (EdU) showed a decrease in fluorescence in MCF-7 vismione E treated cells, due to decreased incorporation of EdU. Visualisation of cell migration, after treatment with 1 µmol/L vismione E, was also done. The results have shown that the centre of the well was freer after 96 h, as compared to the control where the cells filled the well (Girich et al. 2023). *Aspergillus carneus* was isolated from the marine sponge *Agelas oroides* and produced the cytotoxic compound averufanin (**32**). Averufanin-treated breast cancer cells (MCF-7 and T47D) increased in the SubG1 phase as compared to the DMSO control. Morphological changes were also observed, as the cells looked more detached and spherical. The MUSE Cell Analyzer and Annexin V assay also showed an increase in the late apoptotic cells. An increase in the Caspase3/7 activity indicated that averufanin induced apoptosis via the caspase 3/7 cascade. γ-H2Ax staining was also done, and it was observed that accumulation of DNA occurred following treatment with averufanin (Demirel et al. 2023).

**Table 6.** Summary of the mechanism of action of the anticancer compounds.

Compound	Fungus	Cancer cell line	Mechanism of action	Reference
Terrein (**209**)	*Aspergillus terreus* strain PF-26	Human epithelial ovarian cancer cell line, SKOV3	Suppressed cell proliferation Attenuated migration of cells G2/M phase cell cycle arrest Suppressed expression of LIN28 in SKOV3 cells and spheroid Cell death of SKOV3 spheroid	Chen et al. (2014b)
Vismione E (**33**)	*Aspergillus* sp. 1901NT-1.2.2	Human breast cancer MCF-7	Cytotoxic Cell cycle arrest in the G1 phase Decreased number of cells in the first division Decreased cell migration	Girich et al. (2023)
Averufanin (**32**)	*Aspergillus carneus*	Human breast cancer cell lines MCF7, T47D	Increased SubG1 phase Cell morphology changes Accumulation of early and late apoptotic cells Activation of caspase 3/7 cascade Accumulation of DNA damage PARP protein cleavage Dephosphorylation of pGSK3β and phosphorylation of p53 Activation of CDK2-Cyclin A2 cell cycle proteins for DNA Inhibition of Cyclin D1	Demirel et al. (2023)
Wentilactone B (**210**)	*Aspergillus wentii* (EN-48)	Human hepatoma SMMC-7721	Dose-dependent anti-proliferative effect Inhibition of cell colony formation Morphological changes associated with apoptosis Upregulation in expression of Bax and downregulation in expression of Bcl-2 Decreased cell migration through downregulation of CD44 and EGFR proteins	Zhang et al. (2012)
Wentilactone B (**210**)	*Aspergillus wentii* (EN-48)	Human hepatoma SMMC-7721	G2/M phase arrest through increased phosphorylation of p53, cdc2 and cdc25C and in the level of p21. Decrease in the total level of cdc2, cdc25C and cyclin B1 Induction of apoptosis through mitochondrial disruption. Proteolytic cleavage of caspase-9, -7, -3 and PARP. Release of cytochrome *c* (Cyt *c*) from the mitochondria to the cytosol. Decreased levels of antiapoptotic Bcl-xl, -2 and phospho-Bad, and increased expression of Bad and phospho-Bcl-2 Elevated intracellular ROS level Increased phosphorylation of ERK and JNK Binding and activation of Ras and phosphorylation of c-Raf Suppression of tumour growth in mouse xenograft models through upregulation of Ras-GTP, activation of ERK and JNK in xenograft tissues	Zhang et al. (2013)
Fumigaclavine C (**211**)	*Aspergillus fumigatus*	Breast cancer cells MCF-7	Anti-proliferative effect Suppression of cell migration and invasion Inhibition of protein expression of MMP-2 and MMP-9 by inhibiting their mRNAs Blocked signal transduction of MAPK pathway molecules ERK, JNK and p38 Induction of sub-G1 cell population Activation of p53 and p21 proteins and gene levels Down-regulation of CDK2, CDK4, cyclin B1, and cyclin E Morphological changes and DNA damage Down-regulation of PI3K and Akt Down-regulation of anti-apoptotic Bcl-2 and Bcl-xl and up-regulation of pro-apoptotic Bax and Bad levels Increased caspase-3, -8, -9 levels Increased cytochrome C and Apaf-1 in the mitochondria Inhibition of p50 and p65 at transcriptional levels Downregulation of IKK and upstream activation of NF-κB Binding to the cleft of 2w31-Apoptosis and interaction with the key active site residues GLU95 as demonstrated by computational docking studies	Li et al. (2013)
Isosclerone (**212**)	*Aspergillus fumigatus*	Breast cancer cell line MCF-7	Reduction of cancer cell migration Increased sub-G1 fraction of cell Inhibition of protein and gene expression of MMP-2 and MMP-9 Blocked signal transduction of MAPK pathway molecules; ERK, JNK, P38 Down-regulation of CDK2, CDK4, cyclin B1, cyclin E Activation of p53 and p21 protein and gene levels Binding with p53 core DNA-binding domain as demonstrated by docking calculations	Li et al. (2014a)
Isosclerone (**212**)	*Aspergillus fumigatus*	Breast cancer cell line MCF-7	Morphological changes and DNA damage indicating apoptosis DNA fragmentation Downregulation of PI3K, Akt Downregulation of anti-apoptotic Bcl-2 levels and up-regulation of pro-apoptotic Bax levels Expression of caspase-9 protein High expression of caspase-9 and -3 mRNAs Mitochondrial release of cytochrome C and Apaf-1 resulting in processing of caspase-9 causing apoptosis Inhibition of expression of P50 and P65 at transcriptional level Down-regulation of IKK, up-stream-activating of kinases NF-κB Binding to the cleft of 2w31 and 3ITN and interaction with the key active-site residues as shown by docking studies	Li et al. (2014b)
Gliotoxin (**64**)	*Aspergillus* sp.	Human cervical cancer Hela and human chondrosarcoma SW1353	Chromatin condensation and apoptotic bodies Induction of DNA fragmentation Inhibition of cell growth due to induction of apoptosis Depletion of ΔΨm Increased mRNA expression of cyt c, down-regulation of Bcl-2, up-regulation of Bax Increased expression of caspase-3, -8, -9	Nguyen et al. (2014)
Gliotoxin (**64**)	*Aspergillus fumigatus*	Human fibrosarcoma HT1080	Induction of apoptosis Inhibition of NF-κB activation, decrease in amount of nuclear-localised NF-κB-p65 Reduced phosphorylation of IκB-α and increased overall expression of IκB-α Increased ROS levels	Kim and Park (2016)
Demethoxyfumitremorgin C (**213**)	*Aspergillus fumigatus*	Human prostate cancer cells PC3	Induction of apoptosis Disruption of mitochondrial membrane potential Decrease expression of Ras, PI3K, Akt, Bcl-xl and Bcl-2 and increased protein levels of Bax Increased expression of caspase-3, -8, -9 Decreased level of pro-PARP and increase in level of cleaved PARP	Kim et al. (2017)
14-*O*-acetylinsulicolide A (**39**)	*Aspergillus ochraceus* JcmaF17	786-O cells	Enhanced cell population in the G0/G1 phase Induction of late apoptosis after 72 h	Tan et al. (2018)
Asperpyrone A (**214**)	*Aspergillus* sp. XNM-4	PANC-1 cells	Morphological changes such as cell shrinkage and deformation Inhibition of colony formation Induction of cell apoptosis through nuclear pyknosis and chromosome condensation Cell cycle arrest at G0/G1 phase ROS generation Increased ratio of Bax/Bcl-2, activation of caspase-3 and PARP Decreased phosphorylation of PI3K and Akt	Xu et al. (2018)
Acetylapoaranotin (**72**)	*Aspergillus* sp. KMD 901	HCT116 colon cells	Increase in early apoptotic cells Decreased PARP level and increased level of caspase-3, -8, -9 Down-regulation of Bax and Bcl-xl, up-regulation of Bax Tumour reduction observed in *in vivo* xenograft HCT116 nude mouse model	Choi et al. (2011)
Acetylapoaranotin (**72**)	*Aspergillus* sp. AF119	MDA-MB-435	Morphological changes Decrease in cell viability Accumulation of cells of the G2/M phase when cells were treated with 5 µmol/L after 24 h, accumulation of cells of the S phase when the cells were treated with 10 µmol/L after 24 h Increased intracellular ROS level Phosphorylation of H2AX at serine 139 due to DNA double-strand break, resulting in discrete γ-H2AX foci at DNA damage sites	Liu et al. (2012)
(+)-3’,3’-di-(di-methylallyl)-butyrolactone II (**215**), versicolactone B (**106**)	*Aspergillus terreus*	Pancreatic ductal adenocarcinoma cells PANC-1	Morphological features of apoptosis (+)-3’,3’-di-(di-methylallyl)-butyrolactone II (**215**): induction of G2/M and S phase arrest Versicolactone B (**106**): induction of S phase arrest Increased apoptosis rates	Qi et al. (2018)
Versixanthone G (**108**)	*Aspergillus versicolor*	MGC803 cells	Inhibition of Topo I-mediated relaxation of pBR322 DNA Binding with the DNA-Topo I complex as shown by docking results G2/M phase arrest Induction of necrosis but not apoptosis Leakage and disruption of cell membrane	Wu et al. (2018)
(2*R*,4b*R*,6a*S*,12b*S*,12c*S*,14a*S*)-4b-deoxyβ-aflatrem (**216**), (2*R*,4b*S*,6a*S*,12b*S*,12c*R*)-9-isopentenyl paxilline (**217**)	*Aspergillus flavus* OUCMDZ-2205	A549 cells	Cell cycle arrest at S phase PKC-beta inhibition	Sun et al. (2014)
Clavatustide B (**162**)	*Aspergillus clavatus* C2WU	Panc-1, MGC-803, SW-480, WERI-Rb-1, PC3 cells	Delayed G1-S phase cell cycle transition Down-regulation of CCNE2 and up-regulation of FBX031, CYLD	Ye et al. (2014)
Asperindole A (**173**)	*Aspergillus* sp. KMM 4676	22Rv1 cells	Induction of apoptosis S phase cell cycle arrest as well as discrete G2/M phase arrest	Ivanets et al. (2018)
Asperphenin A (**169**)		Colon cancer cells RKO	Accumulation of cells in sub-G1 apoptotic phase, G2/M phase arrest Increases expression of cyclin B1, p-cdc2 (Tyr 15) Inhibition of tubulin polymerisation Activation of p53, pro-apoptotic Bcl2, Bax, Bid, and cleavage of caspase-8, -9, 3 and PARP Increase in intracellular ROS level Enhances effect of irinotecan on cell growth inhibition Suppression of tumour growth in RKO cells-implanted nude mouse xenograft models	Bae et al. (2020)

### 4.2. Aspergillus *associated with algae*

Wentilactone B (**210**) (Figure 7), produced by *Aspergillus wentii* EN-48, caused apoptosis, prevented proliferation and migration of the human hepatoma cells SMMC-7721. An IC_50_ of 31 µmol/L and 19 µmol/L was obtained following treatment for 24 h and 48 h, respectively. Wentilactone B (**210**) did not prevent the growth of normal cells L-02 and Chang cells. When SMMC-7721 cells were treated with 40 µmol/L wentilactone B, colony formation was reduced by 90%. The cells became sparse, round, and small with obscure skeletons. With DAPI staining, bright, condensed chromatin, and fragmented nucleolus could be observed. Moreover, flow cytometry demonstrated an increase from 3% to 40% in apoptotic SMMC-7721 cells as compared to the absence of apoptotic Chang cells. The expression of pro-apoptotic Bax was up-regulated, while the anti-apoptotic Bcl2 was down-regulated after treatment with 20 µmol/L and 40 µmol/L wentilactone B. In the migration assay, the cells migrated into the wound with a decreased distance compared to the untreated ones. CD44 and EGFR proteins were also down-regulated (Zhang et al. 2012). In addition, a dose-dependent cell cycle arrest at the G2/M phase was observed. Phosphorylation of p53, cdc2, and cdc25C was increased while caspase-9, 7 and PARP were activated. It was also observed that cytochrome C was released from the mitochondria to the cytosol. Wentilactone B (**210**) mediated an increase in intracellular ROS levels, phosphorylation of ERK and JNK, leading to apoptosis. It activates Ras, which in turn activates the phosphorylation of MAPK. *In-vivo* studies were also carried out in mouse xenograft models. SMMC-7721 cells were inoculated into nude mice and after day 16 and 20, ~86.4% tumour growth was inhibited after treatment with 20 mg/kg/day wentilactone B. As compared to the positive control 5-FU, which caused a strong decrease in body mass, an increase in body mass was also observed. Histo-immunochemistry revealed that ERK and JNK were also activated *in vivo* (Zhang et al. 2013).

**Figure 8.**
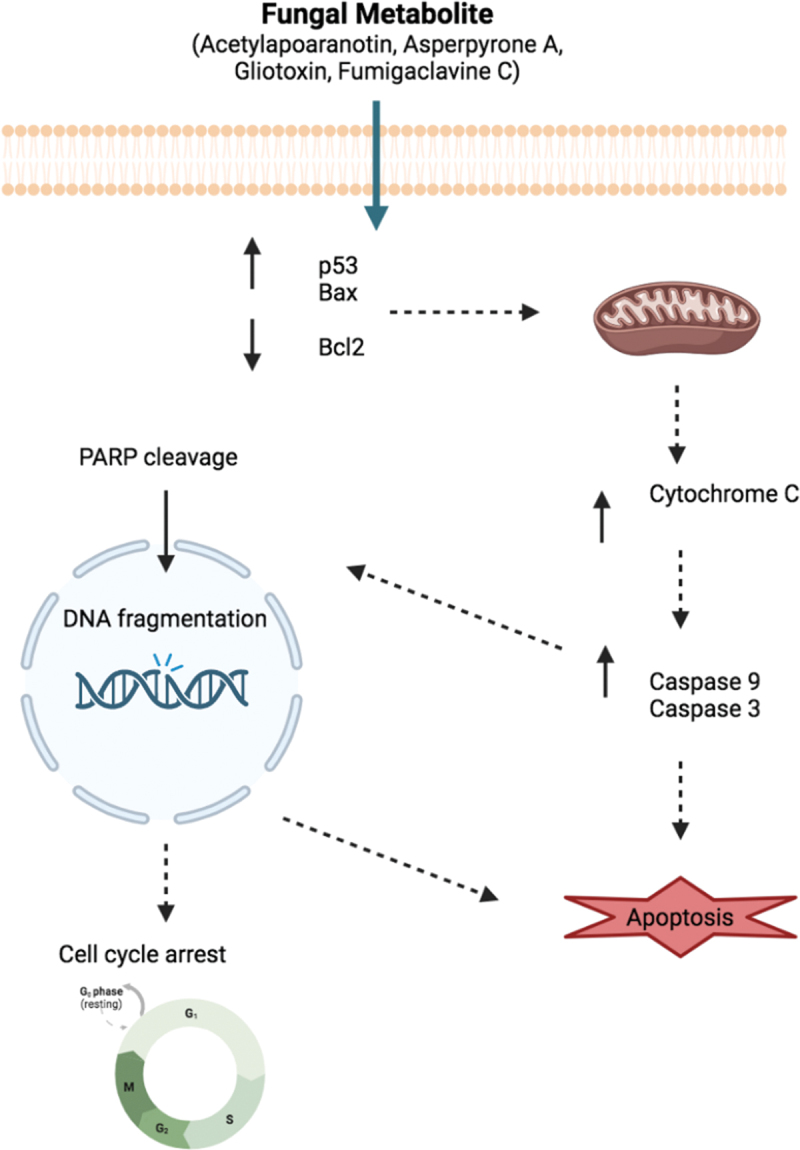
Signaling pathway for fungal metabolites (Acetylapoaranotin, Asperpyrone A, Gliotoxin, and Fumigaclavine) mediated apoptosis in cancer cells.

The marine algal endophyte *Aspergillus fumigatus* produced the compound fumigaclavine C (**211**) (Figure 7). At a concentration of 60 µmol/L, fumigaclavine C blocked the migration and invasion of MCF-7 cells. Western blotting analyses and reverse transcription polymerase chain reaction (RT-PCR) results show inhibition of MMP-2, 9 mRNA. A dose-dependent inhibition of the ERK, JNK, and p38 signalling pathways and induction of the sub-G1 cell population was also encountered. Furthermore, the tumour suppressor factors p21 and p53 were activated, whereas CDK2, CDK4, cyclin B1, and E were down-regulated leading to cell cycle arrest. DNA fragmentation, downregulation of PI3K, AKT, Bcl-2, and Bcl-xl were also obtained after treatment with fumigaclavine C. The levels of caspase-3, 8 and 9 were increased in MCF-7 cells following treatment with fumigaclavine C. This was due to the release of cytochrome C and Apaf-1 from the mitochondria, leading to caspase-9-mediated apoptosis. Fumigaclavine C (**211**) was also associated with suppression of NF-κB and IKK activation. The computational modelling results show that fumigaclavine C binds to the cleft of 2w31-apoptosis, more specifically to the active site residues GLU95 (Li et al. 2013). Figure 8 represents the summarised signalling pathway of fumigaclavine C. Figure 8 was drawn based on the research of Li et al. (2012), Nguyen et al. (2014), and Xu et al. (2018). Another compound, isosclerone (**212**) (Figure 7), was produced by the same fungus. It inhibited MCF-7 cell migration and invasion by increasing the cell percentage in the sub-G1 phase. Western blotting and RT-PCR were then employed to study the effect of isosclerone on protein and gene levels. Inhibition of MMP-2 and MMP-9, ERK, JNK, and p38 MAPK signalling pathway molecules confirmed the anti-proliferative ability of this compound. Moreover, CDK2, CDK4, cyclin B1, and cyclin E were down-regulated and p21 and p53 were activated. Isosclerone was able to bind with p53 core DNA-binding domain (Li et al. 2014a). Isosclerone (**212**) induced apoptosis via DNA fragmentation and morphological changes in the breast cancer cell MCF-7 (Li et al. 2014b). PI3K, Akt, and the anti-apoptotic Bcl-2 were down-regulated while the pro-apoptotic Bax level was up-regulated, leading to induction of apoptosis. Its effect on the caspase-3, -8 and -9 activities was also determined by Western blot analysis. Treatment with isosclerone resulted in a more potent expression of caspase-9 protein as compared to caspase-3 protein. RT-PCR analyses revealed a higher expression of caspase-9 and -3 mRNAs in the MCF-7 cells. Moreover, there was an increase in cytochrome c and Apaf-1 in the cytosol. Expression of P50 and P65 was inhibited at the transcriptional level, IKK was downregulated, up-stream activating kinases of NF-kB. This triggered apoptosis. Docking calculations also revealed that isosclerone binds to the cleft of 2w31 and 3ITN and interacts with the key active-site residues, confirming that it induces apoptosis activity.

Gliotoxin (**64**), isolated from the brown algae endophyte *Aspergillus* sp., was studied for its cytotoxic mechanism on human cervical cancer (HeLa) and human chondrosarcoma (SW1353). Treatment of the cells with this compound resulted in apoptosis by induced DNA fragmentation, chromatin condensation, and disrupted membrane potential. Flow cytometric analyses showed a dose-dependent increase in the number of apoptotic cells. The disruption of ΔΨm shows that gliotoxin is involved in inducing apoptosis. Moreover, it affected protein expression as it activated caspase-3, caspase-8, and caspase-9, up-regulated Bax and cytochrome c (cyt c) and downregulated Bcl-2 (Figure 4). It was noted that glitoxin did not have an effect on p53 protein and gene expression level (Nguyen et al. 2014). Gliotoxin was also isolated from the marine fungus *Aspergillus fumigatus* by Kim and Park (2016). Flow cytometric analyses showed an increase in apoptotic cells (68.7%) following treatment with gliotoxin. It increased ROS levels and inhibited NF-κB activation by preventing phosphorylation and degradation of IκB-α in human fibrosarcoma HT1080 cells.

Demethoxyfumitremorgin C (**213**) (Figure 7), purified from *Aspergillus fumigatus* MFS-150, induced early apoptosis in PC3 cells at a concentration of 0–50 µmol/L, and late-apoptosis at a concentration of 100 µmol/L. There was also a decrease in the mitochondrial potential which blocked the mitochondrial electron transport chain. Moreover, it was able to downregulate the anti-apoptotic proteins Ras, PI3K, Akt, Bcl-xL, and Bcl2, and increase protein levels of Bax. It also upregulated the pro-apoptotic Bax. The level of pro-PARP decreased while PARP cleavage increased, due to an increase in the expression levels of caspase-3, -8 and -9 (Kim et al. 2017). Further investigation on the compound 14-*O*-acetylinsulicolide A (**39**), produced by the fungus *Aspergillus ochraceus* Jcma1F17, was carried out on 786-O cells. Treatment with 14-*O*-acetylinsulicolide A demonstrated cell cycle arrest at the G0/G1 phase at concentration of 1 µmol/L and late apoptosis after 72 h. Insulicolide B also showed weak inhibition of the LPS-induced NF-κB in RAW264.7 cells (Tan et al. 2018).

The compound asperpyrone A (**214**) (Figure 7) was obtained from the *Aspergillus* sp. XNM-4. Morphological changes like cell shrinkage and deformation, nuclear pyknosis, and chromosome condensation were also observed. Inhibition of colony formation, increase in apoptotic population by 40.43 ± 3.27 after treatment with 20 µmol/L asperpyrone A. It can induce apoptosis and cell cycle arrest at the G0/G1 phase in a dose-dependent way. Western blot analysis showed an increase in the proteins Bax/Bcl-2, caspase-3, and PARP. Further studies on asperpyrone A showed induction of apoptosis in PANC-1 by ROS-mediated PI3K/Akt signalling pathway (Figure 4) (Xu et al. 2018).

The secondary metabolites, obtained from the mycelium extract of *Aspergillus unguis* AG 1.1 (G), caused apoptosis in HeLa cells by an increase in apoptotic cells in G_0_ phase. The percentage of cells in the G_0_ phase increased from 9%, 31%, 44%, and 46%, when treated with 10, 25, 50, and 100 µg/mL extract. A decrease in the percentage of cells in the G_1_, S, and G_2_ phases was also observed. Loss of mitochondrial membrane potential and increased ROS production were also obtained after treatment with the extract. ROS production increased from 36.60% to 52.12% at concentration of 10–50 µg/mL (Sajna et al. 2020).

The crude extract of *Aspergillus* sp. caused apoptosis in HeLa cells. After treatment of HeLa cells with 50 µg/mL extract, the percentage dead cell population was 60 ± 1.3 µg/mL. No effect was observed on the normal CHO cells after 48 h, confirming a lack of cytotoxicity against non-cancer cell line. AO/PI staining revealed chromatin condensation and membrane blebbing. The extract arrested the cells at the G2/M phase and decreased cells at the G1 and S phase, producing ROS species, mitochondrial membrane depolarisation, and activating the caspase-3, 7 and 10 pathways (Taritla et al. 2021).

### 4.3. Aspergillus *obtained from sediments*

The three diketopiperazine disulphides, isolated from *Aspergillus* sp. KMD 901, showed an increase in the populations of early apoptotic HCT116 colon cells, compared to the control group. Western blot analyses were performed and PARP decreased, while caspase 3, 9, and 8 were cleaved after 24 h treatment with increasing concentrations of the compounds. Bcl-2 and Bcl-xl expression were down-regulated while Bax was up-regulated. As acetylapoaranotin (**72**) was the more potent compound, *in vivo* xenograft HCT116 nude mouse model was used to assess its in-vitro anti-tumour effect. Acetylapoaranotin (**72**) caused 18.4% and 32.0% tumour reduction volume at 5 and 20 mg/kg as compared to the control group (Choi et al. 2011). After treatment with 3-hydroxyterphyllin, MDA-MB-435 cells showed necrosis, cell distortion, and shrinkage. Moreover, flow cytometric analyses showed an increase in cells at the G2/M phase, following treatment at 5 µmol/L after 24 h. Increase in treatment time caused a decrease in the cells at S and G2/M phase and an accumulation of cells in the G1 phase. When the cells were exposed to 20 and 50 µmol/L of the compound, an increase in intracellular ROS levels was observed (Figure 4). The γ-H2AX foci, which is a sign of DNA double-strand break, were visible after immunofluorescence staining of the treated cells (Liu et al. 2012).

The compounds (+)-3’,3’-di-(di-methylallyl)-butyrolactone II (**215**) (Figure 7) and versicolactone B (**106**), produced by the strain *Aspergillus terreus*, caused apoptosis in PANC-1 cells by nucleus chromatin condensation, nucleus shrinks and DNA fragments. There was also an increase in the fraction of cells in the S phase and G2/M phases by 41.26% and 35.78% following treatment with (+)-3’,3’-di-(di-methylallyl)-butyrolactone II (**215**) at concentration of 25 µmol/L. Versicolactone B caused S phase arrest with an increase of 42.63% cells at 25 µmol/L (Qi et al. 2018). The compounds versixanthone G, H, and K, derived from the fungus *A. versicolor* HDN 1009, were selected to screen for topoisomerase I inhibitory activity. The three compounds were able to inhibit its activity and resulted in nicked DNA. Moreover, versixanthone G (**108**) could inhibit the Topo I-mediated relaxation of pBR322 DNA. Molecular docking suggests that it forms hydrogen bond with the DNA-Topo I. This compound also induced G2/M phase arrest in the MGC803 cells resulting in 47.43% cells following treatment with 25 µmol/L. There was also a decrease in the percentage of cells in the G0/G1 phase. Staining confirmed that versixanthone G (**108**) causes necrosis and disruption of cell membrane integrity (Wu et al. 2018).

### 4.4. Others

The two compounds, (2*R*,4b*R*,6a*S*,12b*S*,12c*S*,14a*S*)-4b-deoxyβ-aflatrem (**216**), (2*R*,4b*S*,6a*S*,12b*S*,12c*R*)-9-isopentenyl paxilline (**217**) (Figure 7), were obtained from *Aspergillus flavus* OUCMDZ-2205. These arrested the cell cycle of A549 in the S phase at a concentration of 10 µmol/L. The first compound also showed PKC-beta inhibition with IC_50_ of 15.6 µmol/L (Sun et al. 2014). Clavatustide B (**162**), obtained from *Aspergillus clavatus* C2WU, caused an accumulation of HepG2 cells in the G1 phase, while the number of cells decreased in the S phase. Regarding the cells Panc-1, MGC-803, SW-480, WERI-Rb-1, and PC3, the G1-S phase was inhibited. Further studies on the genes that regulate the G1-S transition genes were carried out. The gene CCNF2 was down-regulated, while the genes FBX031 and CYLD were up-regulated (Ye et al. 2014). The fungus *Aspergillus* sp. KMM 4676 produced the compound asperindole A (**173**). It induced apoptosis in 22Rv1 cells by S-phase cycle arrest and slight G2/M-phase arrest (Ivanets et al. 2018).

The asperphenin A (**169**), obtained from *Aspergillus* sp., caused an accumulation of RKO cells in the sub-G1 apoptotic phase. Moreover, the percentage of cells in the G2/M phase increased, as well as expression of cyclin B1. The complex cyclin B1/cdc2 showed decreased activity as expression of p-cd2 (Tyr15) was increased after treatment with asperphenin A. The tubulin polymerisation was also inhibited, similar to treatment with vinblastine. Activation of the apoptosis-related proteins p53, Bax, and Bid, and cleavage of caspase-8, 9, 3, and PARP were observed in RKO cells incubated with asperphenin A. Intracellular ROS level also increased in a dose-dependent manner after 24 h. The combined treatment of RKO cells with a mixture of asperphenin A and irinotecan showed growth suppression of the cells. The in-vitro antiproliferative effect was also assessed in nude mouse tumour xenograft models. Tumour growth was reduced by 68.7%, after treatment with 8 mg/kg asperphenin A. Decreased staining of the cellular proliferation marker, Ki-67, ascertained the antitumour activity (Bae et al. 2020). The mechanism of action of *A. flavus*, *A. fumigatus*, and *A. terreus* against HuH-7 and Caco-2 cells was studied by Mohamed et al. (2021). The pro-apoptotic genes Bax and P53 were up-regulated, while Bcl-2 was down-regulated. Caco-2 cells were more sensitive to the *Aspergillus* sp. metabolites, compared to HuH-7, except for *A. terreus*. An up-regulation of cytochrome C was also observed when the cells Caco-2 and HuH-7 were treated with *A. fumigatus* and *A. flavus*. Apoptosis was characterised by cellular DNA accumulation at the G2/M phase and Pre/G1 phase. The highest arrest values were observed with treatment with *A. fumigatus* Caco-2 and Huh-7. Swollen necrotic cells with mixed euchromatin and heterochromatin, abnormal intra-nuclear eosinophilic structures, ruptured cell membranes, shrunken apoptotic cells with peripheral chromatin condensation and dead cells were observed. There were significantly elevated ROS levels in Caco-2 and HuH-7 treated cells.

## 5. Conclusions

Marine fungi from the genus *Aspergillus* have been recovered from different sources around the world, predominantly from sediments. Sponge-derived fungi, especially the *Aspergillus* species, are a prolific source of unique molecular structures and bioactive compounds. Numerous compounds, with unique chemical structures, have successfully been purified and characterised. Nevertheless, very few of these molecules have been profoundly studied in terms of mechanism of action and *in vivo*. Moreover, only one marine-derived *Aspergillus* molecule, Plinabulin, is under clinical trial despite the plethora of anticancer compounds reported in this review. The ability of these compounds to target specific pathways in cancer cell progression makes them ideal candidates for drug development. Therefore, further studies are required on the mechanisms of action of the promising molecules and pre-clinical and clinical studies have to be envisaged. *Aspergillus* species are interesting sources of natural products as they can be cultured *in vitro*, on a large scale. However, the low yield of bioactive metabolites is a major drawback, and some fungi are genetically intractable, making yield improvement problematic. Hence, strain improvement to optimise secondary metabolite production under fermentation conditions has to be addressed.
